# A systematic review and meta-analysis on the efficacy of antibiotic treatment in controlling colibacillosis in broiler production

**DOI:** 10.1371/journal.pone.0326535

**Published:** 2025-07-01

**Authors:** Ronald Vougat Ngom, Helena Cardoso de Carvalho Ferreira, Gaspard Ayissi, Akenghe Tanyienow, Alessandra Piccirillo

**Affiliations:** 1 Department of Animal Production, School of Veterinary Medicine and Sciences, University of Ngaoundere, Ngaoundere, Cameroon; 2 Veterinary Public Health Institute, Vetsuisse Faculty, University of Bern, Bern, Switzerland; 3 University of Liverpool, Faculty of Health and Life Sciences, Institute of Infection, Veterinary and Ecological Sciences, Leahurst, Neston, United Kingdom; 4 Department of Comparative Biomedicine and Food Science, University of Padua, Legnaro, Italy; Suez Canal University, EGYPT

## Abstract

The aim of this study was to evaluate, through a systematic review and meta-analysis, the efficacy of antibiotics in controlling colibacillosis in broiler production, by synthesizing evidence from randomized controlled trials (RCTs). The Cochrane method and the Preferred Reporting Items for Systematic Reviews and Meta-Analyses (PRISMA) guidelines were followed, and the databases CAB abstract, Agricola, PubMed and Web of Science were searched using relevant keywords to identify studies. Eligible studies had to report at least one of the following outcomes: mortality, feed conversion ratio (FCR), condemnations at slaughter and total antibiotic use. Risk of bias (RoB) by outcome of individual study and pairwise meta-analysis by outcome and antibiotic or antibiotics combination, when at least three studies were available, were also conducted. Out of 24,778 articles found in the searched databases, 48 studies were eventually selected. Most of the studies reported mortality as outcome (n = 43) and tested the antibiotic as metaphylactic treatment (n = 41). A total of 47 antibiotics belonging to 18 different classes were tested in the selected studies with enrofloxacin as the most studied. The overall RoB was “some concerns” in 78.2% of studies for mortality outcome and 73.7% for FCR outcome. Meta-analysis showed that doxycycline (0.04; 95% CI 0.02-0.10; P < 0.001) had the highest protective effect against mortality due to colibacillosis, followed by spectinomycin (0.11; 95% CI 0.05-0.26; P < 0.001), enrofloxacin (0.12; 95% CI 0.06-0.25; P < 0.001), lincomycin *plus* spectinomycin (0.22; 95% CI 0.13-0.38; P < 0.001), flumequine (0.25; 95% CI 0.14-0.44; P < 0.001) and oxytetracycline (0.31; 95% CI 0.20-0.50; P < 0.001). However, these results should be interpreted with caution due to the low number of studies included in the meta-analysis and the high variability in the animal models and study designs across RCTs. This review underscores the necessity for continued research to refine treatment strategies of colibacillosis in broiler production in line with the evolving regulatory and public health guidelines related to antibiotic use in animals.

## Introduction

Avian pathogenic *Escherichia coli* (APEC), a group closely related to Extraintestinal Pathogenic *E. coli* (ExPEC) [1,2], can cause extraintestinal diseases (respiratory and systemic infections) in chickens, turkeys, and other avian species, known as avian colibacillosis [3,4]. Several studies have suggested a role of poultry as reservoir for human ExPEC strains [5–7], indicating potential zoonotic implications. For all types and ages of poultry, colibacillosis is a leading cause of morbidity, mortality and reduced productivity, with a significant impact on health, welfare and economics in the poultry industry worldwide [3,4]. APEC can act as primary or secondary pathogens, especially in birds with compromised epithelial and mucosal barriers due to predisposing infections, poor environmental conditions, or other immunological stresses. Disease manifestations in affected birds vary widely, ranging from omphalitis and yolk sac infection in chicks to polyserositis and subcutaneous cellulitis in broilers, as well as salpingitis and peritonitis in breeder layers [4,8]. Health and economic losses due to APEC infections are highly variable and dependent on factors such as disease type, bird category, rearing cycle phase, geographical location [9,10]. However, on-farm mortality, increased feed conversion ratio (FCR) and condemnations at slaughterhouse have been reported to be as high as 20%, 2.7%, and 43%, respectively, leading to economic losses of several millions of euros [11].

Both prevention and control of colibacillosis rely on multiple interventions across the broiler production chain [3,12]. However, biosecurity and hygiene procedures, such as cleaning and disinfection, may fail to eliminate APEC due to its ubiquitous nature and commensal relationship with the bird’s intestinal microflora [8,10]. Vaccination efforts are also hindered by the genetic diversity of pathogenic *E. coli* strains [11,13]. Recent systematic reviews found no conclusive evidence supporting the efficacy of biosecurity measures and vaccination in preventing APEC infections in broilers [14,15]. Antibiotic treatment may be the only option for reducing mortality associated with APEC diseases. Colibacillosis is a major reason for antibiotic use in the broiler industry [16,17], typically administered prophylactically or metaphylactically [18,19]. Common antibiotics used as group treatment (i.e., oral administration through drinking water or feed), belong to the tetracyclines, sulphonamides, β-lactams, fluoroquinolones, and aminoglycosides classes [18–20]. However, the rise of antibiotic and multidrug resistance among APEC strains, coupled with regulatory and public health concerns restricting the antibiotic use in poultry, makes the treatment of colibacillosis increasingly challenging [19,21,22]. The European Food Safety Authority [23] has identified APEC as a relevant antimicrobial resistant pathogen in poultry. The emergence and spread of resistant APEC strains pose serious health and economic challenges for the broiler industry [18,24,25]. Additionally, there is growing concern that poultry may contribute to the selection and dissemination of resistant *E. coli* or resistance genes to humans [6,26,27]. Given the increasing efforts to reduce antibiotic use and the significant implications of antibiotic resistance, there is a pressing need for evidence to identify the most effective antibiotics for treating colibacillosis in broilers.

In 2019, Sargeant et al. [28] conducted a systematic review on the efficacy of antibiotics in controlling colibacillosis in broilers naturally exposed to APEC; however, no conclusive evidence was drawn due to insufficient data. Given the significant health, welfare, and economic impacts of colibacillosis on the poultry production chain a systematic review and meta-analysis, under the framework of the COST Action CA18217 - European Network for Optimization of Veterinary Antimicrobial Treatment (ENOVAT), was conducted to address the following research question: “*In broilers at risk of colibacillosis, does antibiotic treatment versus no antibiotic treatment result in higher FCR/fewer condemnations/lower mortality/total antibiotic use*?”.

## Methods

This systematic review was performed as described by Higgins et al. [29] in the Cochrane Handbook for Systematic Reviews of Interventions. The description is according to the Preferred Reporting Items for Systematic Reviews and Meta-Analyses (PRISMA) 2020 statement [30].

### Protocol and registration

The protocol was developed *a priori* by using the items (headings) recommended in the PRISMA-P guidelines [31]. The protocol was archived in the University of Padua Research Archive institutional repository (https://hdl.handle.net/11577/3460966) and registered online in the Systematic Reviews for Animals and Food (SYREAF) website (https://www.syreaf.org/protocol/).

### Eligibility criteria

The eligibility criteria reported here differ slightly from those established in the original protocol. Criteria for study inclusion were based on the following PICO elements: randomized controlled trials (RCTs) carried out in broiler chickens, including the whole production chain (Population), with natural or experimental exposure to APEC, and treated with any antibiotic (Intervention) used to treat or prevent colibacillosis in chickens (via *in ovo*, injection, feed or water) at doses consistent with therapeutic (metaphylactic) or prophylactic use. Only studies comparing antibiotic treatment to placebo or untreated control groups (Comparator) were considered eligible. Furthermore, only studies published in English, French or Spanish and measuring the results for at least one of the following outcomes were included: mortality, feed conversion ratio (FCR), condemnations at the slaughter, and total antibiotic use (Outcomes). The selection of outcomes was based on prior expert consultation, as described by Paudel et al. [14] and Tilli et al. [15]. No restrictions on geographical location and publication date were imposed.

### Information sources

The databases CAB abstracts (in Ovid) and Agricola (in Proquest) *via* the University of Bern (Switzerland), and PubMed (in MEDLINE) and Web of Science (WOS) through the University of Padua (Italy) were searched using relevant keywords to identify studies. All the databases of WOS were used (Web of science core collection, BIOSIS Citation Index, KCI-Korean Journal Database, Medline, Russian Science Citation Index and SciELO Citation Index) except for Arts & Humanities Citation Index (A&HCI), Conference Proceedings Citation Index-Science (CPCI-S), Conference Proceedings Citation Index-Social Science & Humanities (CPCI-SSH) and Social Sciences Citation Index (SSCI), because their focus was out of the scope of this review. Additionally, the reference list from a previously conducted systematic review on the efficacy of antibiotics to control colibacillosis in broilers [28] was screened.

### Search strategy

The search strategy involved a multi-strand approach that uses a series of searches, with different combinations of concepts, so as to gather all possibly related research and therefore achieve high sensitivity [29]. This strategy included the following concept related to the PICO question: [Broilers] AND [Antimicrobials] AND [Colibacillosis]. The search string formatting was adapted to each selected database interface (S1 Table). The search was conducted twice (March 2022 and November 2023) by using the same search strategy and the same information sources in order to retrieve additional records published in the new timeframe.

Search results from all databases were uploaded in Zotero software (version 6.0.26) and retracted and duplicate citations were removed.

### Study selection

After deduplication, citations found were uploaded in Rayyan software (https://www.rayyan.ai/) for the two-phases screening that was conducted by two independent pairs of reviewers with each pair screening half of the papers. Each pair consisted of one experienced experimented researcher (VN or AP) and one final-year veterinary student (JA or AT). When consensus between reviewers was not reached, an independent experienced reviewer (HF) was consulted. Before the first screening phase (title and abstract), a calibration exercise was carried out by all the reviewers (including the independent reviewer) by screening 120 randomly selected papers. As suggested by Sanguinetti et al. [32], this exercise enables discussion and solves disagreements. For the second phase of the screening (full text), the calibration exercise was carried out on ten randomly selected studies. For this phase, given the low number of papers to screen, each reviewer examined all the studies. Inclusion or exclusion criteria were based on a predefined set. Citations were included when both reviewers of the pair answered ‘Yes’ or ‘Unclear’ to all the questions and excluded when both reviewers responded ‘No’ to any of the screening questions.

The first phase of the screening consisted of evaluating papers retrieved on the basis of their title and abstract. Eligibility of studies was assessed with the following questions:

1. Is the study primary research assessing the use of one or more antibiotic(s) to control colibacillosis in broilers? Yes [Include], No [Exclude], Unclear [Include]
2. Is there a concurrent comparison group? (i.e., controlled with natural or deliberate disease exposure or analytical observational study?) Yes [Include], No [Exclude], Unclear [Include].

Studies meeting these inclusion criteria passed to the next screening phase where the selection process consisted of the full-text screening including the following questions:

1. Is a full text available in English, French or Spanish? Yes [Include], No [Exclude]
2. Is the Population of the study broilers? Yes [Include], No [Exclude], Unclear [Exclude]
3. Is the Intervention of the study the use of antibiotic(s) to control colibacillosis in broilers? Yes [Include], No [Exclude], Unclear [Exclude]
4. Is at least one of mortality, FCR, condemnations at the slaughter due to colibacillosis or indicator of total antibiotic use the Outcome(s) described? Yes [Include], No [Exclude]
5. Is the study design a controlled trial with natural or experimental disease exposure? Yes [Include], No [Exclude, this is a disease challenge study, indicate the antibiotic(s) assessed and herbal extracts]

### Data collection process

A standardized Microsoft Excel® (version 2013) spreadsheet (deviation from the original protocol), created by one author and validated by all the others during the calibration exercise, was used for data extraction. Four independent reviewers performed data extraction from included papers by working *per* pair. Each pair of reviewers extracted data from half of the included papers. Before starting with the data extraction, a calibration exercise was performed by all the reviewers on ten randomly selected papers. Regularly, after extraction of data from five to seven papers, each pair met to validate the data extracted.

### Data items

#### Study characteristics (including population).

Data including general study information (e.g., year of publication, country where the study was conducted, setting and experimental unit, study design and duration) and population (e.g., number and type of flocks, number of birds *per* flock, breed, origin and sex of birds) were extracted from all studies after the full text screening.

#### Intervention details.

Intervention data included a description of the experimental infection of birds with *E. coli* (e.g., serotype, route of infection, age of birds when infected, treatments received before *E. coli* infection, e.g., vaccination, administration of prebiotics) and the antibiotic treatment (i.e., name of the drug, route of administration, frequency and duration of treatment, dose administered, age of birds when the antibiotic was administered, duration between infection and treatment with antibiotic). In addition, data regarding the comparison group (i.e., positive control [PC], i.e., infected and not treated; and negative control [NC], i.e., not infected and not treated) were extracted. The type of treatment (prophylactic or metaphylactic) was also recorded. Prophylactic treatment was considered when the antibiotic was given to birds (by any route) before the infection with *E. coli* and metaphylactic when it was administered to broilers already infected with *E. coli*.

#### Outcomes.

Outcome data included mortality, feed conversion ratio (FCR), condemnations at the slaughter, and total antibiotic use. For all the outcomes, the age of birds when the outcome was measured and the time (start and end) of measurement were recorded.

### Risk of bias assessment

The risk of bias (RoB) assessment deviated from the one proposed in the protocol due to a recent publication [33] of a poultry-specific method to assess the risk of bias. RoB was assessed for all the outcomes extracted using a Microsoft Excel® (version 2013) spreadsheet, and Revman (version 5.4.1) was used to generate the overall RoB based on the data imported from Excel. The following domains of bias were assessed: 1) bias arising from the randomization process (Domain 1); 2) bias due to deviations from the intended interventions (Domain 2); 3) bias due to missing outcome data (Domain 3); 4) bias in the measurement of the outcome (Domain 4); and 5) bias in selection of the reported result (Domain 5). Briefly, each domain of bias is composed of several signaling questions that guide the overall risk of bias. This overall risk of each domain can be then reported as “low risk”, “some concerns”, or “high risk”. For each study, a final overall risk of bias judgment is provided to each outcome based on the results from the five domains. Therefore, a “low risk of bias” outcome would result from all five domains being classified as “low risk”; “some concerns” would result when either one or two domains for that outcome have been classified as “some concerns”; and “high risk of bias” would result from at least three domains being classified as “some concerns” or at least one domain being classified as “high risk”.

### Effect measures

During data extraction, both mortality and condemnations at slaughter were recorded as the number of mortality/condemnation events. Based on these events, Odds Ratios (ORs) were calculated among the populations considered in the trial. The OR was calculated using the standard formula in which the odds of an event in the experimental group were divided by the odds of an event in comparator group. However, according to the Revman Handbook (https://training.cochrane.org/handbook/current/chapter-06), the values of the OR are usually log-transformed before being analyzed. Log transformation makes the confidence intervals (CIs) appear more symmetric, as shown in the graphical displays of performed meta-analyses. The effect measure used for FCR was the mean difference. The mean difference (or more correctly, difference in means) is a standard statistic that measures the absolute difference between the mean values in two groups of a randomized trial. It estimates the amount by which the experimental intervention changes the outcome, on average, compared with the comparator intervention. The meta-analysis for FCR was performed based on the data collected in the original papers, which included the mean value of FCR, the standard deviation, and the number of participants for whom the outcome was measured in each intervention group.

While the OR was calculated using RevMan (version 5.4.1), the mean difference and CIs for continuous data at the intervention arm level were calculated using OpenEpi (https://www.openepi.com/Mean/t_testMean.htm). Effect measures for outcomes were reported with 95% CIs and analyzed using a random effects model, visualized through Forest plots (P < 0.05). The model selection was based on differences in study protocols, which could lead to high heterogeneity. Heterogeneity among studies was assessed using the Cochrane Chi-Square test, with significant heterogeneity considered when the I² value exceeded 50% and P < 0.05. For all effect measures, the control group was always challenged yet untreated. A non-challenged/untreated group was used as comparator only when the challenged/untreated group was not used in the trial.

### Synthesis methods

From the included studies and for each outcome, there were not two or more studies with the same exact intervention (i.e., breed or sex of broilers; type, route and duration of infection; *E. coli* serotype; age of broilers during inoculation/treatment; stress use before inoculation [e.g., use of vaccination or not]; antibiotic class; route of administration, in water/feed/subcutaneous, dosage, frequency and duration of the treatment) and outcome (i.e., duration of the experiment) to permit conducting a formal meta-analysis [29]. However, for antibiotics authorized for use in poultry in Europe, when at least three studies were included, a pairwise meta-analysis was performed for each antibiotic or antibiotics combination with challenged yet untreated group, disregarding the above-mentioned differences (e.g., dosing, administration protocols). Meta-analysis was carried out using the software Review Manager (RevMan, version 5.4.1; https://revman.cochrane.org). The official website of the European Union (EU) on medicines authorized for use in animals in the EU/EEA (https://medicines.health.europa.eu/veterinary/en) was consulted to categorize antibiotics.

### Sensitivity assessment

Sensitivity analysis was performed for the meta-analysis results that showed a high and significant level of heterogeneity among the studies (I^2 ^> 50%, P ≤ 0.05). Lower heterogeneity was achieved by first removing studies with the high risk of bias. Afterwards, if the heterogeneity was still high, studies that presented differences in the population and intervention during the experiments were removed as studies of low methodological quality can alter the result interpretation [34].

### Reporting bias across studies

According to the protocol, publication bias would only be carried out if more than ten studies are included in the meta-analysis, and this was not the case for any of the antibiotics considered in this study.

## Results

### Study selection

**Fig 1:**
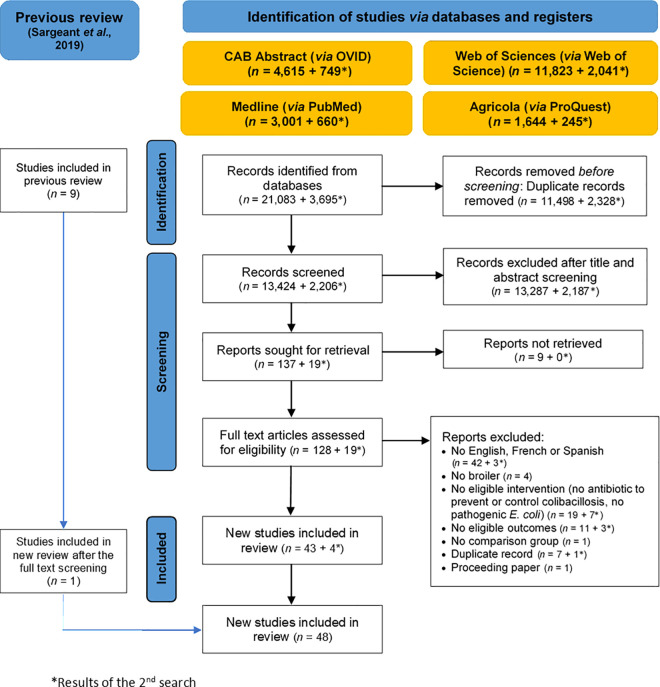
Flow diagram showing the selection process for the systematic review on the efficacy of antibiotics to control colibacillosis in broilers according to PRISMA guidelines.

Out of 24,778 citations found in the four databases, 13,826 retracted and duplicate citations were found in Zotero. After removal of these, 15,630 were uploaded in Rayyan for the two-phases screening (Fig 1). Title and abstract screening excluded 15,474 citations and only 156 were included. Nine articles were non-retrievable; hence, 147 articles were eligible for the full text screening. Forty-seven studies reporting at least one eligible outcome were selected after the full text screening (Fig 1). In addition, after screening the reference list of a previously conducted review [28], one met our inclusion criteria [35] and included in our review with a total of 48 papers eligible for the final analyses. All the included studies were RCTs with deliberate disease exposure except for one, which reported natural disease exposure [35]. The list of all studies analyzed during the full text screening, along with the decisions made by the reviewers, is provided in S2 Table.

### Study characteristics

Table 1 summarizes the general characteristics for the 48 eligible studies included in the systematic review. Studies were conducted between the 1960s (n = 2; 4.2%) and the 2020s (n = 11; 22.9%). The location where the study was conducted was not reported in ten (20.8%) studies, while the remaining were conducted in Europe (n = 13; 27.1%), America (n = 9; 18.7%), Asia (n = 8; 16.7%), and Africa (n = 8; 16.7%). Most of the studies (n = 46; 95.8%) were in English, two in French. Trials were conducted mainly in experimental (n = 26; 54.2%) or commercial flocks (n = 18; 37.5%), or both (n = 1; 2.1%); however, the setting and the experimental unit were not mentioned in more than half of the studies, i.e., 36 (75.0%) and 21 (43.8%) studies, respectively. The number of flocks included in the trials ranged between one and 56 (excluding the negative control groups), and all but one trial included negative control groups in the experiment. Trials started between one and 40 days of age and lasted from six to 62 days (mean = 27 days); the most common were one day (n = 10; 20.8%) and 28 days (n = 8; 16.7%), respectively.

**Table 1:** General characteristics of the 48 studies included in the systematic review investigating the efficacy of antibiotics to control colibacillosis in broiler production.

Study	Country	Setting	Experimental unit	Bird origin	Breed	Sex	Bird age (days)	No. of trials	No. of flocks	Study duration (days)	Eligible outcomes
Hamdy & Blanchard [36]	NR	NR	NR	Commercial	NR	Male	6	1	9	21	Mortality
Hebert & Chang [37]	NR	Research center	Pen	NR	NR	NR	14	11	73	13	Mortality, Condemnations at slaughter
Zolli & Polewaczyk [38]	USA	NR	Pen	Experimental	White Rock	Male	1	6	30	28	Mortality, Condemnations at slaughter
Sieiro & Meier [39]	Switzerland	NR	Cage	Commercial	Hubbard	NR	21	7	70	27	Mortality
George et al. [40]	USA	Research center	Cage, pen	Experimental	H&N (breeders)	Male	13	3	12	7	Mortality
Hamdy et al. [41]	USA	NR	Battery	Experimental	NR	Male	1	1	11	10	Mortality
Goren et al. [42]	Netherlands	NR	Cage	Experimental	Ross	NR	17	4	25	28	Mortality
Cracknell et al. [35]	Italy, Greece, Jordan	Farm	Pen	Commercial	Hubbard, Golden comet, Cobb	NR	27-39	12	24	15	Mortality
Karmy et al. [43]	Egypt	NR	NR	Commercial	NR	NR	28	2	9	35	Mortality
Goren et al. [44]	Netherlands	NR	Cage	Experimental	Ross	NR	17	2	9	28	Mortality
Goren et al. [45]	Netherlands	NR	Cage	Experimental	Ross, Hybro	Both	17	3	13	10	Mortality
Goren et al. [46]	Netherlands	NR	Cage	Experimental	Hybro	Male	17	4	12	11	Mortality
Labarthe et al. [47]	France	NR	Cage	Experimental	Leghorn	NR		1	4	NR	Mortality
Phukan et al. [48]	India	NR	NR	Experimental	NR	NR	3-21	4	37	6	Mortality
Mogenet et al. [49]	France	NR	Cage	Experimental	Ross	Both	21	1	3	28	Mortality
Charleston et al. [50]	NR	NR	Cage	Commercial	Ross	Both	14	1	8	35	Mortality, FCR
Sumano et al. [51]	NR	Farm	NR	Commercial	NR	NR	21	1	3	NR	Mortality
Ashraf et al. [52]	Pakistan	NR	NR	Commercial	NR	NR	1	1	3	NR	Mortality
Chansiripornchai & Sasipreeyajan [53]	NR	NR	NR	Experimental	Arbor Acres	Both	18-40	3	9	28-48	Mortality, FCR
Fernandez et al. [54]	Spain	University	NR	Commercial	Hybro	NR	5	2	10	7	Mortality
Sarközy et al. [55]	Hungary	NR	Pen	NR	Ross	NR	10	1	3	10	Mortality
Glisson et al. [56]	USA	NR	Pen	Experimental	Ross	Unsexed	21	1	4	42	Mortality, FCR
Huff et al. [57]	NR	NR	Pen	Commercial	Cobb 500	Male	21	2	6	21	Mortality
Velkers et al. [58]	Netherlands	Research center	Cage, pen	Commercial	Cobb	NR	1	4	12	15	Mortality
Phad et al. [59]	NR	NR	NR	Commercial	NR	NR	8	1	4	NR	Mortality
Masoud et al. [60]	NR	NR	NR	Commercial	Cobb	NR	15	1	4	33	Mortality, FCR
Chansiripornchai [61]	Thailand	NR	NR	Commercial	Arbor Acres	Both	21	1	8	7	Mortality, FCR
Abd El-Ghany & Madian [62]	NR	NR	NR	Commercial	Hubbard	Both	14	1	3	42	Mortality, FCR
Dheilly et al. [63]	France	NR	Cage	Experimental	Leghorn SPF	Both	14-16	1	6	62	Mortality
Hassan et al. [64]	NR	NR	Cage	Commercial	Arbor Acres	NR	7	1	3	42	Mortality
Peek et al. [65]	Netherlands	NR	Pen	Experimental	NR	Male	8	4	12	15-36	Mortality
Abd El-Ghany & Ismail [66]	Egypt	NR	NR	Experimental	Hubbard	NR	14	1	4	42	Mortality, FCR
Shalaby et al. [67]	Egypt	NR	NR	NR	NR	NR	15	1	4	40	Mortality
Lezzar et al.^*^ [68]	Algeria	NR	NR	Both	ISA 15 & unidentified	NR	NR	1	6	38	NE
Foltz et al. [69]	USA	University	Cage	Experimental	NR	Male	7	1	3	28	Mortality, FCR
El-Keredy et al. [70]	Egypt	NR	Cage	Experimental	NR	NR	1	1	5	35	Mortality, FCR
Roth et al. [71]	USA	Company	Pen	Experimental	Ross	Male	1	1	3	38	Mortality, FCR
Aguilar-Urquizo et al. [72]	Mexico	NR	Pen	Experimental	Ross 308	Male	25	1	3	40	Mortality
Ahmad et al. [73]	Pakistan	NR	NR	Commercial	Hubbard	NR	35	1	4	24	Mortality, FCR
Tarazi et al. [74]	Jordan	University	Cage	Experimental	Hubbard classical	NR	21	1	6	28	Mortality
ul Haq et al. [75]	Pakistan	NR	NR	Experimental	Ross 500	NR	NR	1	3	35	Mortality, FCR
El Hammed et al. [76]	Egypt	NR	NR	Experimental	Cobb 500	NR	19-21	1	3	21	Mortality
Jahanian et al. [77]	Iran	NR	Pen	Experimental	Ross	Female	7	1	3	42	Mortality, FCR
Ahmad et al. [78]	Pakistan	University	NR	Experimental	Hubbard	NR	20	1	3	32	Mortality, FCR
Eid et al. [79]	Egypt	NR	NR	Commercial	NR	NR	1	1	4	21	Mortality, FCR
El-Tahawy et al. [80]	Egypt	NR	NR	Experimental	Cobb	NR	1	1	3	28	FCR
Gunawardana et al.^*^ [81]	Canada	University	Pen	Experimental	Ross 308	NR	1	1	2	16	Mortality
Helmy et al. [82]	USA	University	NR	Commercial	Cobb	NR	1	1	3	42	Mortality, FCR

* These papers were excluded from the meta-analysis because they did not present outcomes in an extractable form; NR = not reported; FCR = feed conversion ratio; NE = not extractable.

### Results of individual studies

Out of 48 studies included in the systematic review, 25 (52.1%) reported only mortality as outcome, three (6.3%) only FCR. Furthermore, no study reported only condemnations at slaughter, or reported total antibiotic use as outcome (Table 1). Sixteen (33.3%) studies reported both mortality and FCR, two (4.2%) both mortality and condemnations at slaughter, and none reported the three outcomes. Most of the studies (n = 41; 85.4%) tested the antibiotic as metaphylactic (i.e., for the control of colibacillosis), two (4.2%) as prophylactic (i.e., for the prevention of colibacillosis), in four (8.3%) both treatments were tested, and in one (2.1%) it was not possible to retrieve the information. Two studies [68,81] did not present outcomes in an extractable form; therefore, they were excluded from the downstream analyses.

Among the 46 studies from which outcomes data were extracted, one (n = 28; 60.9%) to ten (n = 1; 2.2%) different antibiotics were evaluated as metaphylactic or prophylactic treatment of colibacillosis in broiler production (Tables 2–4). In total, 47 different antibiotics, alone (n = 43; 93.5%) or in combination (n = 4; 8.7%), of 18 different classes were studied with enrofloxacin (n = 10; 21.7%), oxytetracycline (n = 6; 13%), doxycycline (n = 5; 10.9%), spectinomycin (n = 4; 8.7%), and lincomycin *plus* spectinomycin (n = 4; 8.7%) as the most common. Nine different combinations of antibiotics were tested in twelve studies (26.1%); however, in one study [51] only the antibiotic class (cephalosporins *plus* fluoroquinolones) was indicated.

**Table 2:** Intervention arms and results for mortality outcome in studies included in the systematic review on the efficacy of antibiotics to control colibacillosis in broiler production.

Study	Intervention	No. birds	Mortality	Comparator	No. birds	Mortality	OR (95%CI)
Hamdy & Blanchard [36]	Lincomycin + Spectinomycin (1:2), 2 gm/gal, water, 3 days	15	4	NC	15	10	0.18 (0.04, 0.87)
	Lincomycin + Spectinomycin (1:2), 2 gm/gal, water, 7 days	15	4	NC	15	10	0.18 (0.04, 0.87)
	Lincomycin + Spectinomycin (1:2), 2 gm/gal, water, 10 days	15	4	NC	15	10	0.18 (0.04, 0.87)
	Lincomycin + Spectinomycin (1:2), 3 gm/gal, water, 3 days	15	5	NC	15	10	0.25 (0.05, 1.14)
	Lincomycin + Spectinomycin (1:2), 3 gm/gal, water, 7 days	15	3	NC	15	10	0.13 (0.02, 0.66)
	Lincomycin, 2 gm/gal, water, 7 days	15	8	NC	15	10	0.57 (0.13, 2.50)
	Spectinomycin, 2 gm/gal, water, 7 days	15	4	NC	15	10	0.18 (0.04, 0.87)
	Tylosin, 2 gm/gal, water, 5 days	15	8	NC	15	10	0.57 (0.13, 2.50)
Hebert & Chang [37]	Furazolidone, 0.005%, feed, 13 days	120	45	PC	140	81	0.44 (0.27, 0.72)
				NC	140	0	169.34 (10.29, 2787.58)
	Furazolidone, 0.00825%, feed, 13 days	120	36	PC	140	81	0.31 (0.19, 0.52)
				NC	140	0	121.38 (7.35, 2003.51)
	Furazolidone, 0.011%, feed, 13–15 days	5805	702	PC	5805	3734	0.08 (0.07, 0.08)
				NC	5805	0	1598.26 (99.85,25583.95)
	Furazolidone, 0,022%, feed, 13 days	1420	48	PC	1420	977	0.02 (0.01, 0.02)
				NC	1420	0	100.39 (6.18, 1629.67)
	Furazolidone, 100 g/ton, feed, 13 days	90	9	PC	90	63	0.05 (0.02, 0.11)
				NC	90	0	21.10 (1.21, 368.23)
	Furazolidone, 200 g/ton, feed, 10 days	30	2	PC	30	14	0.08 (0.02, 0.41)
				NC	30	0	5.35 (0.25, 116.31)
	Chlortetracycline, 100 g/ton, feed, 13 days	90	48	PC	90	63	0.49 (0.27, 0.90)
				NC	90	0	206.55 (12.44, 3430.13)
	Chlortetracycline, 500 g/ton, feed, 10 days	30	9	PC	30	14	0.49 (0.17, 1.41)
				NC	30	0	26.95 (1.49, 488.33)
	Oxytetracycline, 200 g/ton, feed, 10–13 days	120	47	PC	120	77	0.36 (0.21, 0.61)
				NC	120	0	155.75 (9.46, 2564.86)
	Bacitracin zinc, 50 g/ton, feed, 13 days	90	45	PC	90	63	0.43 (0.23, 0.79)
				NC	90	0	181.00 (10.90, 3005.39)
	Bacitracin zinc, 500 g/ton, feed, 10 days	30	11	PC	30	14	0.66 (0.24, 1.86)
				NC	30	0	35.97 (2.00, 645.93)
	Penicillin + Streptomycin, 30 g/ton + 150 g/ton, feed, 13 days	120	73	PC	120	77	0.87 (0.51, 1.46)
				NC	120	0	372.92 (22.64, 6141.16)
	Penicillin procaine, 50 g/ton, feed, 13 days	90	58	PC	90	63	0.78 (0.42, 1.45)
				NC	90	0	325.80 (19.57, 5424.16)
	Penicillin procaine, 100 g/ton, feed, 10 days	30	15	PC	30	14	1.14 (0.41, 3.15)
				NC	30	0	61.00 (3.42, 1088.58)
	Erythromycin, 92.5 g/ton, feed, 13 days	90	43	PC	90	63	0.39 (0.21, 0.72)
				NC	90	0	165.76 (9.98, 2752.46)
	Erythromycin, 185 g/ton, feed, 10 days	30	16	PC	30	14	1.31 (0.47, 3.60)
				NC	30	0	69.41 (3.89, 1239.18)
	Bacitracin methylene disalicyclate, 100 g/ton, feed, 13 days	90	48	PC	90	63	0.49 (0.27, 0.90)
				NC	90	0	206.55 (12.44, 3430.13)
	Bacitracin methylene disalicyclate, 200 g/ton, feed, 10 days	30	16	PC	30	14	1.31 (0.47, 3.60)
				NC	30	0	69.41 (3.89, 1239.18)
	Tylosin, 0.0132%, water, 13 days	30	20	PC	90	63	0.86 (0.35, 2.07)
				NC	90	0	353.38 (19.89, 6277.98)
Zolli & Polewaczyk [38]	Spectinomycin, 2.5 mg, subcutaneous, 1 day	580	43	NC	578	18	2.49 (1.42, 4.37)
				PC	583	249	0.11 (0.08, 0.15)
	Spectinomycin, 5 mg, subcutaneous, 1 day	580	32	NC	578	18	1.82 (1.01, 3.28)
				PC	587	249	0.08 (0.05, 0.12)
	Spectinomycin, 10 mg, subcutaneous, 1 day	578	16	NC	578	18	0.89 (0.45, 1.75)
				PC	587	249	0.04 (0.02, 0.07)
Sieiro & Meier [39]	Sulphachloropyridazine, 5 g/kg, feed, 3–5 days	80	0	NC	80	0	Not estimable
				PC	80	9	0.05 (0.00, 0.82)
	Sulphachloropyridazine, 4 g/kg, feed, 5 days	20	0	NC	20	0	Not estimable
				PC	20	2	0.18 (0.01, 4.01)
	Sulphachloropyridazine, 3.5 g/kg, feed, 5 days	40	0	NC	20	0	Not estimable
				PC	20	2	0.09 (0.00, 2.00)
	Sulphachloropyridazine, 2.75 g/kg, feed, 5 days	20	0	NC	20	0	Not estimable
				PC	20	2	0.18 (0.01, 4.01)
	Sulphachloropyridazine, 2.6 g/kg, feed, 5 days	20	0	NC	20	0	Not estimable
				PC	20	2	0.18 (0.01, 4.01)
	Sulphachloropyridazine, 2.5 g/kg, feed, 5 days	20	0	NC	20	0	Not estimable
				PC	20	2	0.18 (0.01, 4.01)
	Sulphachloropyridazine, 1.25 g/kg, feed, 5 days	20	0	NC	20	0	Not estimable
				PC	20	2	0.18 (0.01, 4.01)
	Sulphachloropyridazine, 3 g/l, water, 6 days	80	8	NC	80	0	18.88 (1.07, 332.84)
				PC	80	5	1.67 (0.52, 5.33)
	Sulphachloropyridazine, 2 g/l, water, 1–5 days	160	0	NC	160	0	Not estimable
				PC	160	10	0.04 (0.00, 0.77)
	Sulphachloropyridazine, 1.6 g/l, water, 5 days	20	0	NC	20	0	Not estimable
				PC	20	4	0.09 (0.00, 1.78)
	Sulphachloropyridazine, 1.4 g/l, water, 5 days	20	0	NC	20	0	Not estimable
				PC	20	4	0.09 (0.00, 1.78)
	Sulphachloropyridazine, 1.3 g/l, water, 5 days	20	0	NC	20	0	Not estimable
				PC	20	4	0.09 (0.00, 1.78)
	Sulphachloropyridazine, 1.04 g/l, water, 5 days	20	0	NC	20	0	Not estimable
				PC	20	4	0.09 (0.00, 1.78)
	Sulphachloropyridazine, 1 g/l, water, 1–5 days	100	0	NC	100	0	Not estimable
				PC	100	5	0.09 (0.00, 1.58)
	Sulphachloropyridazine, 0.9 g/l, water, 5 days	20	0	NC	20	0	Not estimable
				PC	20	4	0.09 (0.00, 1.78)
	Sulphachloropyridazine, 0.5 g/l, water, 5 days	20	0	NC	20	0	Not estimable
				PC	20	2	0.18 (0.01, 4.01)
	Sulphachloropyridazine, 50 mg AI/kg, parenteral, 2 days	20	0	NC	20	0	Not estimable
				PC	20	3	0.12 (0.01, 2.53)
	Sulphachloropyridazine, 100 mg AI/kg, parenteral, 2 days	20	0	NC	20	0	Not estimable
				PC	20	3	0.12 (0.01, 2.53)
	Furazolidone, 400 ppm, 5 days	20	0	NC	20	0	Not estimable
				PC	20	2	0.18 (0.01, 4.01)
	Furazolidone, 3.6 g/kg, feed, 3–5 days	60	0	NC	60	0	Not estimable
				PC	60	7	0.06 (0.00, 1.06)
	Furazolidone, 2.88 g/kg, feed, 5 days	20	0	NC	20	0	Not estimable
				PC	20	2	0.18 (0.01, 4.01)
	Chloramphenicol + Furaltadone, 2.625 g/l, water, 6 days	80	18	NC	80	0	47.66 (2.82, 806.25)
				PC	80	5	4.35 (1.53, 12.40)
	Chloramphenicol + Furaltadone, 2 g/l, water, 3–5 days	80	1	NC	80	0	3.04 (0.12, 75.69)
				PC	80	11	0.08 (0.01, 0.63)
	Chloramphenicol + Furaltadone, 1.6 g/l, water, 5 days	20	NR	NC	20	0	NA
				PC	20	4	NA
	Sulphapyrazole, 50 mg AI/kg, parenteral, 2 days	20	1	NC	20	0	Not estimable
				PC	20	3	0.12 (0.01, 2.53)
	Sulphapyrazole, 100 mg AI/kg, parenteral, 2 days	20	0	NC	20	0	Not estimable
				PC	20	3	0.12 (0.01, 2.53)
	Oxytetracycline, 50 mg AI/kg, parenteral, 2 days	20	0	NC	20	0	Not estimable
				PC	20	3	0.12 (0.01, 2.53)
	Oxytetracycline, 100 mg AI/kg, parenteral, 2 days	20	0	NC	20	0	Not estimable
				PC	20	3	0.12 (0.01, 2.53)
	Polymyxin methane sulphonate, 50 mg AI/kg, parenteral, 2 days	20	0	NC	20	0	Not estimable
				PC	20	3	0.12 (0.01, 2.53)
	Polymyxin methane sulphonate, 100 mg AI/kg, parenteral, 2 days	20	0	NC	20	0	Not estimable
				PC	20	3	0.12 (0.01, 2.53)
George et al. [40]	Doxycycline, 3 g/6l, water, 3 days	120	2	PC	120	40	0.03 (0.01, 0.14)
	Chlortetracycline, 4.5 g/6l, water, 3 days	120	14	PC	120	40	0.26 (0.13, 0.52)
	Lincomycin + Spectinomycin, 4.6 g/6l, water, 3 days	120	11	PC	120	40	0.20 (0.10, 0.42)
Hamdy et al. [41]	Lincomycin, 1.25 mg/kg, subcutaneous, 1 day	50	42	PC	50	28	4.13 (1.61, 10.56)
				NC	40	0	405.00 (22.63, 7247.01)
	Lincomycin, 2.5 mg/kg, subcutaneous, 1 day	50	24	PC	50	28	0.73 (0.33, 1.59)
				NC	40	0	74.89 (4.36, 1284.89)
	Lincomycin, 5 mg/kg, subcutaneous, 1 day	50	25	PC	50	28	0.79 (0.36, 1.73)
				NC	40	0	81.00 (4.72, 1389.66)
	Lincomycin, 10 mg/kg, subcutaneous, 1 day	50	18	PC	50	28	0.44 (0.20, 0.99)
				NC	40	0	46.11 (2.68, 794.46)
	Spectinomycin, 5 mg/kg, subcutaneous, 1 day	50	6	PC	50	28	0.11 (0.04, 0.30)
				NC	40	0	11.83 (0.65, 216.70)
	Lincomycin + Spectinomycin, 1.25 + 5 mg/kg, subcutaneous, 1 day	50	13	PC	50	28	0.28 (0.12, 0.64)
				NC	40	0	29.16 (1.67, 507.86)
	Lincomycin + Spectinomycin, 2.5 + 5 mg/kg, subcutaneous, 1 day	50	7	PC	50	28	0.13 (0.05, 0.34)
				NC	40	0	13.97 (0.77, 252.43)
	Lincomycin + Spectinomycin, 5 + 5 mg/kg, subcutaneous, 1 day	50	7	PC	50	28	0.13 (0.05, 0.34)
				NC	40	0	13.97 (0.77, 252.43)
	Lincomycin + Spectinomycin, 10 + 5 mg/kg, subcutaneous, 1 day	50	2	PC	50	28	0.03 (0.01, 0.15)
				NC	40	0	4.18 (0.19, 89.48)
Goren et al. [42]	Sulfaclozine, 1 mg/l, water, 5 days	32^*^	0	NC	32^*^	4	0.10 (0.01, 1.89)
	Sulphadimidine, 2 mg/l, water, 5 days	32^*^	0	NC	32^*^	4	0.10 (0.01, 1.89)
	Sulphadimidine, 250 mg/l, water, 4 days	63^$^	14	NC	63^$^	16	0.84 (0.37, 1.91)
	Sulphaquinoxaline, 187 mg/l, water, 4 days	63^$^	9	NC	63^$^	16	0.49 (0.20, 1.21)
	Sulphaquinoxaline, 200 mg/l, water, 4 days	32^*^	0	NC	32^*^	2	0.19 (0.01, 4.07)
	Sulphaquinoxaline, 300 mg/l, water, 4 days	32^*^	0	NC	32^*^	2	0.19 (0.01, 4.07)
	Trimethoprim, 66 mg/l, water, 4 days	32^*^	0	NC	32^*^	2	0.19 (0.01, 4.07)
	Trimethoprim, 99 mg/l, water, 4 days	32^*^	0	NC	32^*^	2	0.19 (0.01, 4.07)
	Trimethoprim, 122 mg/l, water, 4 days	32^*^	1	NC	32^*^	4	0.23 (0.02, 2.14)
	Trimethoprim, 330 mg/l, water, 4 days	32^*^	0	NC	32^*^	4	0.10 (0.01, 1.89)
	Trimethoprim, 660 mg/l, water, 4 days	32^*^	0	NC	32^*^	4	0.10 (0.01, 1.89)
	Trimethoprim, 1320 mg/l, water, 4 days	32^*^	0	NC	32^*^	4	0.10 (0.01, 1.89)
	Sulphaquinoxaline + Trimethoprim, 133 mg/l, water, 4 days	32^*^	0	NC	32^*^	2	0.19 (0.01, 4.07)
	Sulphaquinoxaline + Trimethoprim, 266 mg/l, water, 4 days	32^*^	0	NC	32^*^	2	0.19 (0.01, 4.07)
	Sulphaquinoxaline + Trimethoprim, 300 mg/l, water, 4 days	63	4	NC	32^*^	16	0.07 (0.02, 0.23)
	Sulphaquinoxaline + Trimethoprim, 399 mg/l, water, 4 days	32^*^	0	NC	32^*^	2	0.19 (0.01, 4.07)
	Sulphadiazine + Trimethoprim, 280 mg/l, water, 4 days	63^$^	3	NC	63^$^	16	0.15 (0.04, 0.53)
Cracknell et al. [35]	Apramycin, 500 mg/l, water, 5 days	18408	433	PC	820	480	0.02 (0.01, 0.02)
	Apramycin, 250 mg/l, water, 5 days	19393	13270	PC	900	181	8.61 (7.29, 10.16)
	Apramycin, 125 mg/l, water, 5 days	10272	2154	PC	3400	142	6.09 (5.11, 7.25)
Karmy et al. [43]	Flumequine, 3 mg/kg, water, 4 days	40	1	PC	10	1	0.23 (0.01, 4.05)
	Flumequine, 6 mg/kg, water, 4 days	40	0	PC	10	1	0.08 (0.00, 2.07)
	Flumequine, 9 mg/kg, water, 4 days	40	0	PC	10	1	0.08 (0.00, 2.07)
	Flumequine, 12 mg/kg, water, 4 days	40	0	PC	10	1	0.08 (0.00, 2.07)
Goren et al. [44]	Sulphadimidine, 1000 ppm, water, 4 days	50	0	PC	50	1	0.33 (0.01, 8.21)
	Sulphadimidine, 500 ppm, water, 4 days	50	0	PC	50	1	0.33 (0.01, 8.21)
	Sulphadimidine, 350 ppm, water, 4 days	25	0	PC	25	1	0.32 (0.01, 8.25)
	Sulphadimidine, 250 ppm, water, 4 days	50	0	PC	50	1	0.33 (0.01, 8.21)
Goren et al. [45]	Doxycycline, 25 ppm, water, 4 days	32^*^	0	PC	32^*^	2	0.19 (0.01, 4.07)
	Doxycycline, 50 ppm, water, 4 days	32^*^	0	PC	32^*^	2	0.19 (0.01, 4.07)
	Doxycycline, 100 ppm, water, 4 days	32^*^	0	PC	32^*^	2	0.19 (0.01, 4.07)
	Doxycycline, 200 ppm, water, 4 days	32^*^	1	PC	32^*^	2	0.48 (0.04, 5.62)
	Doxycycline, 400 ppm, water, 4 days	32^*^	0	PC	32^*^	1	0.32 (0.01, 8.23)
	Doxycycline, 600 ppm, water, 4 days	32^*^	0	PC	32^*^	1	0.32 (0.01, 8.23)
	Doxycycline, 800 ppm, water, 4 days	32^*^	0	PC	32^*^	1	0.32 (0.01, 8.23)
	Doxycycline, 1000 ppm, water, 4 days	32^*^	0	PC	32^*^	1	0.32 (0.01, 8.23)
	Flumequine, 100 ppm, water, 4 days	32^*^	1	PC	32^*^	2	0.48 (0.04, 5.62)
	Tetracycline, 1000 ppm, water, 4 days	32^*^	0	PC	32^*^	1	0.32 (0.01, 8.23)
Goren et al. [46]	Lincomycin + Spectinomycin, 0.127 + 0.255 g/l, water, 5 days	33^#^	9	PC	33^#^	8	1.17 (0.39, 3.54)
	Lincomycin + Spectinomycin, 0.255 + 0.511 g/l, water, 5 days	65^£^	10	PC	65^£^	10	1.00 (0.39, 2.59)
	Lincomycin + Spectinomycin, NC, water, 5 days	33^#^	1	PC	33^#^	1	1.00 (0.06, 16.69)
	Spectinomycin, 1 g/l, water, 5 days	33^#^	0	PC	33^#^	0	Not estimable
	Spectinomycin, 2 g/l, water, 5 days	33^#^	0	PC	33^#^	0	Not estimable
	Spectinomycin, 0.513 g/l, water, 5 days	65^£^	5	PC	65^£^	16	0.26 (0.09, 0.75)
	Spectinomycin, 1.026 g/l, water, 5 days	65^£^	1	PC	65^£^	16	0.05 (0.01, 0.37)
	Neomycin, 1 g/l, water, 5 days	33^#^	0	PC	33^#^	0	Not estimable
Labarthe et al. [47]	Ceftriaxone, 50 mg/kg, intravenous, NR	29	0	PC	19	4	0.06 (0.00, 1.16)
	Cefotaxime, 50 mg/kg, intravenous, NR	28	1	PC	19	4	0.14 (0.01, 1.36)
	Cefotaxime, 100 mg/kg, intravenous, NR	17	0	PC	19	4	0.10 (0.00, 1.98)
Phukan et al. [48]	Chloramphenicol, 200 mg/kg, intraperitoneal, 5 days	40	12	PC	40	40	0.01 (0.00, 0.10)
				NC	40	0	35.53 (2.02, 624.72)
				Saline	10	0	9.21 (0.50, 169.74)
	Nalidixic acid, 100 mg/kg, subcutaneous, 5 days	40	6	PC	40	40	0.00 (0.00, 0.04)
				NC	40	0	15.26 (0.83, 280.72)
				Saline	10	0	3.96 (0.21, 76.22)
	Sulfamethoxazole + Trimethoprim, 2 g/100 birds, subcutaneous, 5 days	40	12	PC	40	40	0.01 (0.00, 0.10)
				NC	40	0	35.53 (2.02, 624.72)
				Saline	10	0	9.21 (0.50, 169.74)
Mogenet et al. [49]	Amoxicillin, 20 mg/kg, water, 6 days	1154	350	PC	48	24	0.44 (0.24, 0.78)
	Flumequine, 12 mg/kg, water, 6 days	571	124	NC	2400	24	27.46 (17.54, 43.01)
Charleston et al. [50]	Enrofloxacin, 9.5 mg/kg, water, 3 days	90	10	NC	90	0	23.61 (1.36, 409.32)
				PC	90	39	0.16 (0.08, 0.36)
	Enrofloxacin, 11.3 mg/kg, water, 3 days	90	6	NC	90	0	13.92 (0.77, 250.94)
				PC	90	39	0.09 (0.04, 0.24)
	Danofloxacin, 5.2 mg/kg, water, 3 days	90	17	NC	90	0	43.10 (2.55, 728.75)
				PC	90	39	0.30 (0.16, 0.60)
	Danofloxacin, 5.7 mg/kg, water, 3 days	90	15	NC	90	0	37.16 (2.19, 631.37)
				PC	90	39	0.26 (0.13, 0.52)
	Sarafloxacin, 8.2 mg/kg, water, 5 days	90	24	NC	90	0	66.68 (3.98, 1116.28)
				PC	90	39	0.48 (0.25, 0.89)
	Sarafloxacin, 8.8 mg/kg, water, 5 days	90	18	NC	90	0	46.19 (2.74, 779.48)
				PC	90	39	0.33 (0.17, 0.63)
Sumano et al. [51]	Cephalosporin + Fluoroquinolone, 3 mg/kg, bolus, 1 day	45	0	PC	45	45	0.00 (0.00, 0.01)
	Enrofloxacin, 10 mg/kg, bolus, 1 day	45	6	PC	45	45	0.00 (0.00, 0.03)
Ashraf et al. [52]	Gentamycin, 2 mg/bird, intrayolk, 1 day	35	2	PC	35	17	0.06 (0.01, 0.31)
				NC	35	0	5.30 (0.25, 114.47)
Chansiripornchai & Sasipreeyajan [53]	Sarafloxacin, 5 mg/kg, water, 3 days	118	18	PC	112	66	0.13 (0.07, 0.23)
				NC	76	0	28.16 (1.67, 474.74)
	Sarafloxacin, 10 mg/kg, water, 3 days	16	0	PC	16	9	0.02 (0.00, 0.47)
				NC	16	0	Not estimable
Fernandez et al. [54]	Fosfomycin, 50 mg/l, water, 4 days	15	0	NC^¥^	30	0	Not estimable
				PC	30	3	0.25 (0.01, 5.23)
	Fosfomycin, 100 mg/l, water, 4 days	15	1	NC^¥^	30	0	6.31 (0.24, 164.56)
				PC	30	3	0.64 (0.06, 6.76)
	Fosfomycin, 200 mg/l, water, 4 days	15	0	NC^¥^	30	0	Not estimable
				PC	30	3	0.25 (0.01, 5.23)
	Fosfomycin, 50 mg/l, feed, 4 days	15	9	NC^¥^	30	0	89.15 (4.59, 1732.91)
				PC	30	8	4.13 (1.11, 15.32)
	Fosfomycin, 100 mg/l, feed, 4 days	15	8	NC^¥^	30	0	69.13 (3.58, 1336.70)
				PC	30	8	3.14 (0.86, 11.50)
	Fosfomycin, 200 mg/l, feed, 4 days	15	8	NC^¥^	30	0	69.13 (3.58, 1336.70)
				PC	30	8	3.14 (0.86, 11.50)
Sarközy et al. [55]	Norfloxacin, 100 mg/l, water, 5 days	15	0	PC	10	4	0.05 (0.00, 1.00)
	Norfloxacin, 15 mg/kg, water, 5 days	15	0	PC	10	4	0.05 (0.00, 1.00)
Glisson et al. [56]	Enrofloxacin, 25 ppm, water, 3 days	20	0	PC	20	18	0.00 (0.00, 0.07)
	Oxytetracycline, 400 mg/kg, water, 6 days	20	1	PC	20	18	0.01 (0.00, 0.07)
	Sulfadimethoxine, 1,875 mg/kg, water, 6 days	20	1	PC	20	18	0.01 (0.00, 0.07)
Huff et al. [57]	Enrofloxacin, 50 ppm, water, 7 days	40	1	PC	40	27	0.01 (0.00, 0.10)
				NC	40	2	0.49 (0.04, 5.60)
Velkers et al. [58]	Doxycycline, 250 mg/l, water, 5 days	96	0	NC	96	0	Not estimable
				PC	95	45	0.01 (0.00, 0.10)
Phad et al. [59]	Colistin, 100 mg/kg, feed, 35 days	100	0	NC	50	NR	NA
				PC	50	11	NA
Masoud et al. [60]	Apramycin, 25 mg/kg, water, 3 days	100	14	NC	50	0	16.93 (0.99, 289.92)
				PC	50	22	0.21 (0.09, 0.46)
Chansiripornchai [61]	Oxytetracycline, 30 mg/kg, water, 3 days	60	30	PC	20	14	0.43 (0.15, 1.26)
				NC	20	0	41.00 (2.37, 708.76)
	Enrofloxacin, 10 mg/kg, water, 3 days	60	0	PC	20	14	0.00 (0.00, 0.07)
				NC	20	0	Not estimable
Abd El-Ghany & Madian [62]	Sarafloxacin, 5 mg/kg, water, 3 days	50	5	NC	50	0	12.21 (0.66, 226.97)
				PC	50	19	0.18 (0.06, 0.54)
Dheilly et al. [63]	Amoxicillin, 10 mg/kg, water, 5 days	62	26	NC	60	0	87.85 (5.20, 1485.42)
				PC	60	13	2.61 (1.18, 5.78)
	Sulfadimethoxine + Trimethoprim, 28 + 6 mg/kg, water, 5 days	57	5	NC	60	0	12.68 (0.68, 234.70)
				PC	60	13	0.35 (0.12, 1.05)
	Oxytetracycline, 20 mg/kg, water, 3 days	58	4	NC	60	0	9.99 (0.53, 189.85)
				PC	60	13	0.27 (0.08, 0.88)
	Enrofloxacin, 10 mg/kg, water, 5 days	58	3	NC	60	0	7.63 (0.39, 151.06)
				PC	60	13	0.20 (0.05, 0.73)
Hassan et al. [64]	Oxytetracycline, 1 g/kg, feed, NR	50	5	PC	50	17	0.22 (0.07, 0.64)
				NC	50	5	1.00 (0.27, 3.69)
Peek et al. [65]	Doxycycline, 125 mg/l, water, 4 days	28	2	PC	30	8	0.21 (0.04, 1.10)
				NC	30	0	5.75 (0.26, 125.30)
	Doxycycline, 250 mg/l, water, 4 days	90	0	PC	90	19	0.02 (0.00, 0.34)
				NC	90	1	0.33 (0.01, 8.20)
Abd El-Ghany & Ismail [66]	Ciprofloxacin, 5 mg/kg, water, 5 days	40	2	NC	40	0	5.26 (0.24, 113.11)
				PC	40	16	0.08 (0.02, 0.37)
Shalaby et al. [67]	Enrofloxacin, 10 mg/kg, NR, 5 days	20	0	NC	20	0	Not estimable
				PC	20	12	0.02 (0.00, 0.31)
Foltz et al. [69]	Bacitracin methylene disalicyclate, 50 g/ton, feed, 28 days	72	5	NC	72	7	0.69 (0.21, 2.29)
				PC	72	0	11.81 (0.64, 217.75)
El-Keredy et al. [70]	Neomycin, 1 mg/l, water, 5 days	50	10	NC	50	0	26.19 (1.49, 460.45)
				PC	50	20	0.38 (0.15, 0.92)
Roth et al. [71]	Ampicillin, 100 mg/1000l, water, 5 days	400	14	NC	400	12	1.17 (0.54, 2.57)
				PC	400	17	0.82 (0.40, 1.68)
Ahmad et al. [73]	Enrofloxacin + Colistin, 1 ml/4l + 1 mg/10l, water, 5 days	35	2	PC	35	14	0.09 (0.02, 0.44)
	Enrofloxacin, 1 ml/4l, water, 5 days	35	8	PC	35	14	0.44 (0.16, 1.26)
	Colistin, 1 mg/10l, water, 5 days	35	6	PC	35	14	0.31 (0.10, 0.94)
Aguilar-Urquizo et al. [72]	Enrofloxacin, 10%, NR	10	0	PC	10	2	0.16 (0.01, 3.85)
				NC	10	0	Not estimable
Tarazi et al. [74]	Doripenem, NR, intravenous, 7 days	30	17	PC	30	16	1.14 (0.41, 3.17)
				NC	30	0	79.07 (4.42, 1413.18)
	Cefepime, NR, intravenous, 7 days	30	1	PC	30	16	0.03 (0.00, 0.25)
				NC	30	0	3.10 (0.12, 79.23)
	Tigecycline, NR, intravenous, 7 days	30	15	PC	30	16	0.88 (0.32, 2.41)
				NC	30	0	61.00 (3.42, 1088.58)
	Tetracycline, NR, intravenous, 7 days	30	27	PC	30	16	7.88 (1.96, 31.68)
				NC	30	0	479.29 (23.68, 9700.89)
El Hammed et al. [76]	Difloxacin, 10 mg/kg, water, NR	50	4	NC	50	0	9.77 (0.51, 186.52)
				PC	50	15	0.20 (0.06, 0.67)
Ahmad et al. [78]	Ciprofloxacin, 20 mg/kg, water, 5 days	50	7	NC	50	1	7.98 (0.94, 67.46)
				PC	50	14	0.42 (0.15, 1.15)
Eid et al. [79]	Doxycycline, 1 g/l, water, 5 days	15	1	NC	15	NR	NA
				PC	15	4	0.20 (0.02, 2.02)
Helmy et al. [82]	Sulfadimethoxine, 495.323 mg/L, water, 7 days	75	3	NC^¥^	75	1	3.08 (0.31, 30.34)

NA: Not applicable; NR: Not reported; NC: Negative control (no infection/no treatment); PC: Positive control (infection/no treatment);

¥ : Not infected and not treated (with antibiotic), broilers received the placebo;

* : the number of animals in the trial was between 30 and 33;

# : the number of animals in the trial was between 30 and 35;

$ : the number of animals in the trial was between 60 and 66;

£ : the number of animals in the trial was between 60 and 70.

**Table 3:** Intervention arms and results for FCR outcome in studies included in the systematic review on the efficacy of antibiotics to control colibacillosis in broiler production.

Study	Intervention	No. Birds (# Flocks)	FCR	SD	Comparator	No. Birds (# Flocks)	FCR	SD	Mean Difference^a^ (95%CI)
George et al. [40]	Doxycycline, 3 g/6l, in water, 3 days	120 (12)	2.02	0.21	PC	90 (12)	2.52	0.57	−0.50 (−0.54, −0.46)
	Chlortetracycline, 4.5 g/6l, in water, 3 days	117 (12)	2.17	0.28	PC	90 (12)	2.52	0.57	−0.35 (−0.48, −0.22)
	Lincomycin + Spectinomycin, 4.6 g/6l, water, 3 days	120 (12)	2.23	0.39	PC	90 (12)	2.52	0.57	−0.29 (−0.43, −0.15)
Cracknell et al. [35]	Apramycin, 500 mg/l, water, 5 days	8700 (3)	19.57	0.20	PC	2,900 (3)	2,94	0.13	16.63 (16.62, 16.64)
	Apramycin, 250 mg/l, water, 5 days	8700 (3)	10.63	9.3	PC	2,900 (3)	2.94	0.13	7.69 (7.18, 8.20)
	Apramycin, 125 mg/l, water, 5 days	8700 (3)	8.2	6.19	PC	2,900 (3)	2.94	0.13	5.26 (0.77, 5.75)
Charleston et al. [50]	Enrofloxacin, 9.5 mg/kg, water, 3 days	90 (1)	2.1	0.1	NC	90 (1)	2.2	0.20	−0.10 (−0.15, 0.50)
					PC	90 (1)	2.80	0.6	−0.70 (−0.83, −0.57)
	Enrofloxacin, 11.3 mg/kg, water, 3 days	90 (1)	2.2	0.2	NC	90 (1)	2.2	0.20	0 (−0.04, 0.04)
					PC	90 (1)	2.80	0.6	−0.60 (−0.73, −0.47)
	Danofloxacin, 5.2 mg/kg, water, 3 days	90 (1)	2.1	0.30	NC	90 (1)	2.2	0.20	−0.10 (−0.17, −0.02)
					PC	90 (1)	2.80	0.6	−0.70 (−0.84, −0.56)
	Danofloxacin, 5.7 mg/kg, water, 3 days	90 (1)	2.1	0.30	NC	90 (1)	2.2	0.20	−0.10 (−0.17, −0.02)
					PC	90 (1)	2.80	0.6	−0.70 (−0.84, −0.56)
	Sarafloxacin, 8.2 mg/kg, water, 5 days	90 (1)	2.6	0.6	NC	90 (1)	2.2	0.20	0.40 (0.27, 0.53)
					PC	90 (1)	2.80	0.6	−0.20 (−0.38, −0.02)
	Sarafloxacin, 8.8 mg/kg, water, 5 days	90 (1)	2.2	0.3	NC	90 (1)	2.2	0.20	0 (−0.07, 0.07)
					PC	90 (1)	2.80	0.6	−0.60 (−0.74, −0.46)
Chansiripornchai & Sasipreeyajan [53]	Sarafloxacin, 5 mg/kg, water, 3 days	118 (3)	2.9	NR	PC	36 (1)	NR	0.04	NA
					NC	76 (1)	2.8	NR	NA
	Sarafloxacin, 10 mg/kg, water, 3 days	16 (1)	2.8	NR	PC	16 (1)	NR	0.04	NA
					NC	16 (1)	2.8	NR	NA
Glisson et al. [56]	Enrofloxacin, 25 ppm, in water, 3 days	20 (1)	1.81	NR	PC	20 (1)	1.86	0,08	NA
	Oxytetracycline, 400 mg/kg, in water, 6 days	20 (1)	1.90	NR	PC	20 (1)	1,86	0,06	NA
	Sulfadimethoxine, 1,875 mg/kg, in water, 6 days	20 (1)	1.84	NR	PC	20 (1)	1.86	0,08	NA
Masoud et al. [60]	Apramycin, 25 mg/kg, in water, 3 days	100 (2)	1.80	0.06	PC	50 (1)	2.41	NR	NA
					NC	50 (1)	2.41	NR	NA
Chansiripornchai [61]	Oxytetracycline, 30 mg/kg, in water, 3 days	60 (3)	4.20	1.24	PC	20 (1)	5.53	7.81	−1.33 (−5.0, 2.34)
					NC	20 (1)	1.67	0.13	2.53 (2.20, 2.86)
	Enrofloxacin, 10 mg/kg, in water, 3 days	60 (3)	1.78	0.06	PC	20 (1)	5.53	7.81	−3.75 (−7.40, −0.09)
					NC	20 (1)	1.67	0.13	0.11 (0.05, 0.17)
Abd El-Ghany & Madian [62]	Sarafloxacin, 5 mg/kg, in water, 3 days	50 (1)	1.89	NR	PC	50 (1)	2.40	NR	NA
					NC	50 (1)	1.78	NR	NA
Abd El-Ghany & Ismail [66]	Ciprofloxacin, 5 mg/kg, in water, 5 days	40 (1)	2.20	NR	PC	40 (1)	2.40	NR	NA
					NC	40 (1)	1.93	NR	NA
Foltz et al. [69]	Bacitracin methylene disalicyclate, 50 g/ton, in feed, 28 days	72 (1)	1.54	NR	NC	72 (1)	1.53	NR	NA
					PC	72 (1)	1.6	NR	NA
El-Keredy et al. [70]	Neomycin, 1 mg/l, in water, 5 days	50 (1)	1.70	0.01	PC	50 (1)	1.90	0.02	−0.20 (−0.21, −0.19)
					NC	50 (1)	1.7	0.01	0 (−0.03, 0.03)
Roth et al. [71]	Ampicillin, 100 mg/1000l, in water, 5 days	400 (1)	1.62	0.03	PC	400 (1)	1.68	0.06	−0.06 (−0.07, −0.05)
					NC	400 (1)	1.64	0.03	−0.02 (−0.02, −0.01)
Ahmad et al. [73]	Enrofloxacin + Colistin, 1 ml/4l + 1 mg/10l, in water, 5 days	35 (1)	2.45	NR	PC	35 (1)	2.89	NR	NA
	Enrofloxacin, 1 ml/4l, in water, 5 days	35 (1)	2.51	NR	PC	35 (1)	2.89	NR	NA
	Colistin, 1 mg/10l, in water, 5 days	35 (1)	2.65	NR	PC	35 (1)	2.89	0.31	NA
Ul Haq et al. [75]	Enrofloxacin, 10 mg/kg, NR	80 (1)	1.49	0.04	PC	80 (1)	1.44	0.02	0.05 (0.04, 0.06)
					NC	80 (1)	1.41	0.01	0.08 (0.07, 0.09)
Jahanian et al. [77]	Virginiamycin, NR, in feed, NR	12 (1)	1.58	NR	NC	24 (2)	1.66	NR	NA
					PC	24 (2)	1.91	NR	NA
Ahmad et al. [78]	Ciprofloxacin, 20 mg/kg, in water, 5 days	50 (1)	2.0	NR	PC	50 (1)	2.15	NR	NA
					NC	50 (1)	1.9	NR	NA
Eid et al. [79]	Doxycycline, 1 g/l, in water, 5 days	15 (1)	1.33	0.02	NC	15 (1)	1.54	0.03	0 (−0.01, 0.01)
					PC	15 (1)	1.95	10.03	−062 (−6.17, 4.49)
El-Tahawy et al. [80]	Cefquinome, 2 mg/kg, intramuscular, 3 days	50 (1)	1.51	0.20	PC	50 (1)	1.93	0.02	−0.42 (−0.48, −0.36)
					NC	50 (1)	1.47	0.01	0.04 (−0.02, 0.10)
Helmy et al. [82]	Sulfadimethoxine, 495.323 mg/L, in water, 7 days	75 (1)	1.81	0.38	NC^¥^	75 (1)	1.80	0.43	0.88 (−0.12, 0.14)
					NC	75 (1)	1.65	NR	NA

FCR: Feed Conversion Ratio; NA: Not applicable; NR: Not reported; NC: Negative control (No infection/no treatment); PC: Positive control (Infection/no treatment);

¥ : Not infected and not treated (with antibiotic) but broilers received a placebo.

**Table 4:** Intervention arms and results for condemnations at slaughter outcome in studies included in the systematic review on the efficacy of antibiotics to control colibacillosis in broiler production.

Study	Intervention	No. birds	No. flocks	Outcome	Comparator	No. birds	No. flocks	Outcome	OR (95%CI)
Hebert & Chang [37]	Furazolidone, 0.005%, feed, 13 days	180	3	93	PC	180	3	142	0.29 (0.18, 0.45)
					NC	180	3	0	385.75 (23.67, 6286.20)
	Furazolidone, 0.00825%, feed, 13 days	180	3	131	PC	180	3	142	0.72 (0.44, 1.16)
					NC	180	3	0	959.02 (58.62, 15689.26)
	Furazolidone, 0.011%, feed, 13–15 days	5845	6	2144	PC	5845	6	4215	0.22 (0.21, 0.24)
					NC	5845	6	0	6773.29 (423.40, 108356.21)
	Furazolidone, 0,022%, feed, 13 days	1460	4	328	PC	1460	4	1102	0.09 (0.08, 0.11)
					NC	1460	4	0	847.28 (52.83, 13589.28)
	Furazolidone, 100 g/ton, feed, 13 days	90	3	28	PC	90	3	32	0.82 (0.44, 1.52)
					NC	90	3	0	82.54 (4.95, 1377.15)
	Furazolidone, 200 g/ton, feed, 10 days	30	1	5	PC	30	1	15	0.20 (0.06, 0.66)
					NC	30	1	0	13.16 (0.69, 249.48)
	Chlortetracycline, 100 g/ton, feed, 13 days	90	3	36	PC	90	3	32	1.21 (0.66, 2.21)
					NC	90	3	0	121.22 (7.29, 2015.25)
	Chlortetracycline, 500 g/ton, feed, 10 days	30	1	11	PC	30	1	15	0.58 (0.21, 1.62)
					NC	30	1	0	35.97 (2.00, 645.93)
	Oxytetracycline, 200 g/ton, feed, 10–13 days	120	4	59	PC	120	4	47	1.50 (0.90, 2.51)
					NC	120	4	0	233.16 (14.17, 3835.52)
	Bacitracin zinc, 50 g/ton, feed, 13 days	90	3	36	PC	90	3	32	1.21 (0.66, 2.21)
					NC	90	3	0	121.22 (7.29, 2015.25)
	Bacitracin zinc, 500 g/ton, feed, 10 days	30	1	14	PC	30	1	15	0.88 (0.32, 2.41)
					NC	30	1	0	53.61 (3.00, 956.98)
	Penicillin + Streptomycin, 30 g/ton + 150 g/ton, feed, 13 days	120	4	70	PC	120	4	32	3.85 (2.24, 6.63)
					NC	120	4	0	336.45 (20.44, 5538.02)
	Penicillin procaine, 50 g/ton, feed, 13 days	90	3	45	PC	90	3	32	1.81 (1.00, 3.29)
					NC	90	3	0	181.00 (10.90, 3005.39)
	Penicillin procaine, 100 g/ton, feed, 10 days	30	1	21	PC	30	1	15	2.33 (0.81, 6.73)
					NC	30	1	0	138.05 (7.62, 2501.17)
	Erythromycin, 92.5 g/ton, feed, 13 days	90	3	33	PC	90	3	32	1.05 (0.57, 1.93)
					NC	90	3	0	105.45 (6.34, 1754.90)
	Erythromycin, 185 g/ton, feed, 10 days	30	1	21	PC	30	1	15	2.33 (0.81, 6.73)
					NC	30	1	0	138.05 (7.62, 2501.17)
	Bacitracin methylene disalicyclate, 100 g/ton, feed, 13 days	90	3	39	PC	90	3	32	1.39 (0.76, 2.53)
					NC	90	3	0	138.83 (8.36, 2306.33)
	Bacitracin methylene disalicyclate, 200 g/ton, feed, 10 days	30	1	19	PC	30	1	15	1.73 (0.62, 4.84)
					NC	30	1	0	103.43 (5.76, 1857.21)
	Tylosin, 0.0132%, water, 13 days	90	3	25	PC	90	3	32	0.70 (0.37, 1.31)
					NC	90	3	0	70.47 (4.21, 1178.48)
Zolli & Polewaczyk [38]	Spectinomycin, 2.5 mg, subcutaneous, 1 day	578	6	34	NC	578	6	24	1.44 (0.84, 2.47)
					PC	587	6	379	0.03 (0.02, 0.05)
	Spectinomycin, 5 mg, subcutaneous, 1 day	580	6	56	NC	578	6	24	2.47 (1.51, 4.04)
					PC	587	6	379	0.06 (0.04, 0.08)
	Spectinomycin, 10 mg, subcutaneous, 1 day	580	6	96	NC	578	6	24	4.58 (2.88, 7.28)
					PC	587	6	379	0.11 (0.08, 0.14)

NC: Negative control (No infection/no treatment), PC: Positive control (Infection/no treatment).

### Risk of bias within studies

**Fig 2:**
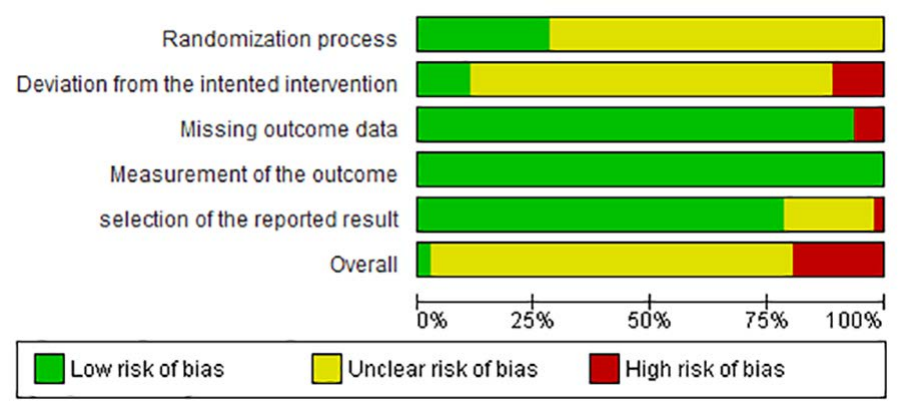
Risk of bias results for each one of the five domains for the mortality outcome.

**Fig 3:**
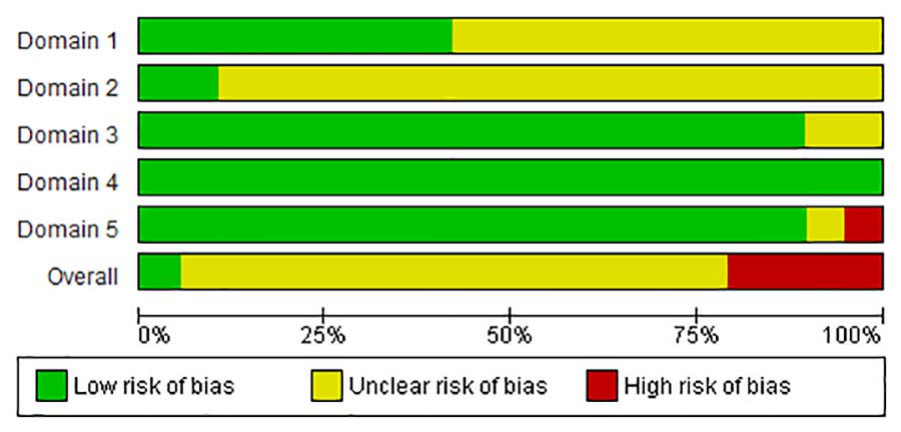
Risk of bias results for each one of the five domains for the FCR outcome.

**Fig 4:**
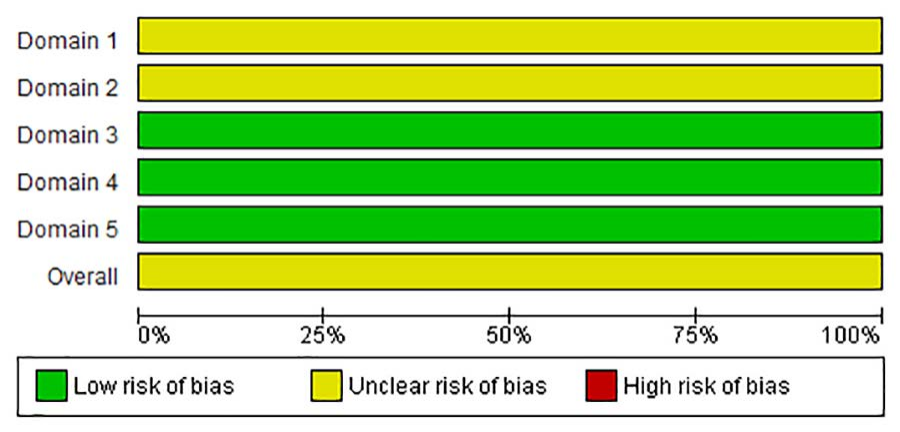
Risk of bias results for each one of the five domains for the condemnations at the slaughter outcome.

For the 43 studies including mortality as outcome, the overall RoB was “high” (n = 9; 19.6%), “some concerns” (n = 33; 78.2%), and “low” (n = 1; 2.2%). For the RoB due to deviation from the intended intervention (domain 2), only 10.9% (n = 5) of the studies was assessed as “low” (Fig 2). For the FCR outcome (n = 19 studies), the overall RoB was “high” for 21.0% (n = 4) of the studies, “some concerns” for 73.7% (n = 14) and “low” for 5.3% (n = 1). For this outcome, domain 2 also represented the area of bias with only two (10.5%) studies with a “low” risk (Fig 3). For both mortality and FCR, the overall RoB due to measurement of the outcome (domain 4) was assessed as “low”. The overall RoB of the two studies [37,38] with the condemnations at the slaughter as outcome was assessed as “some concerns” because both RCTs were classified with “some concerns” in domains 1 and 2 (Fig 4). For all outcomes, the main reason for bias was the lack of information about researchers’ awareness of broilers assigned the intervention during the experiment.

### Synthesis of results

**Fig 5:**
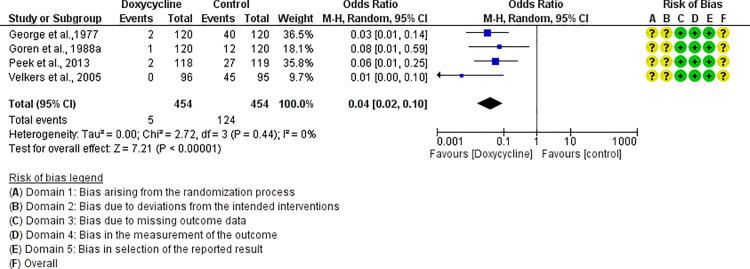
Forest plot of doxycycline efficacy to control mortality caused by colibacillosis in broilers.

**Fig 6:**
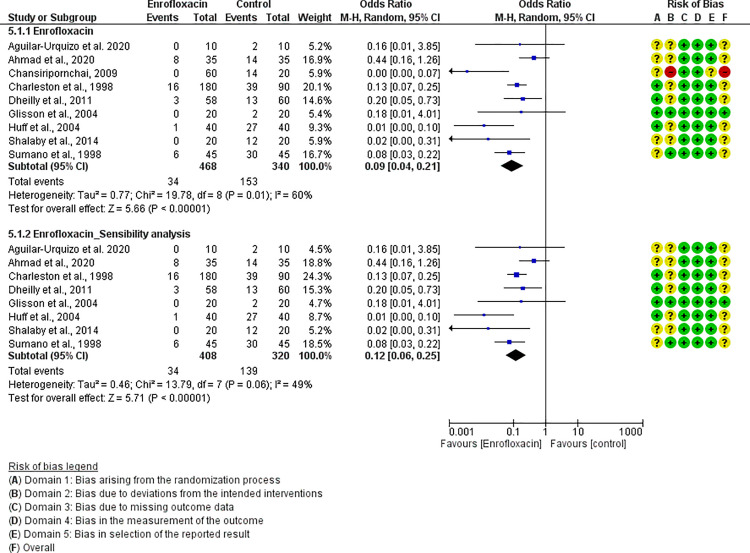
Forest plot of enrofloxacin efficacy to control mortality caused by colibacillosis in broilers.

**Fig 7:**
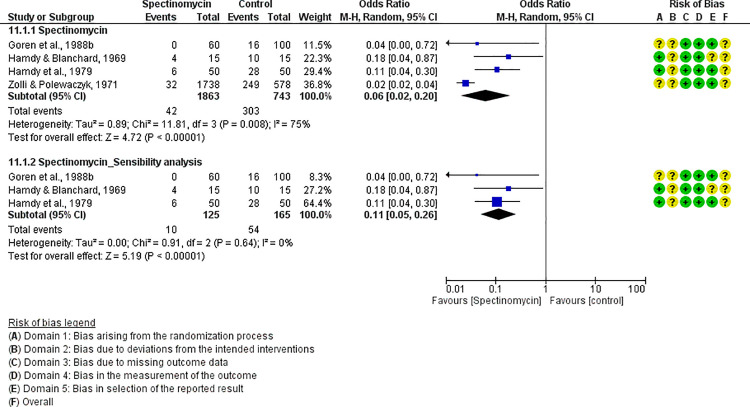
Forest plot of spectinomycin efficacy to control mortality caused by colibacillosis in broilers.

**Fig 8:**
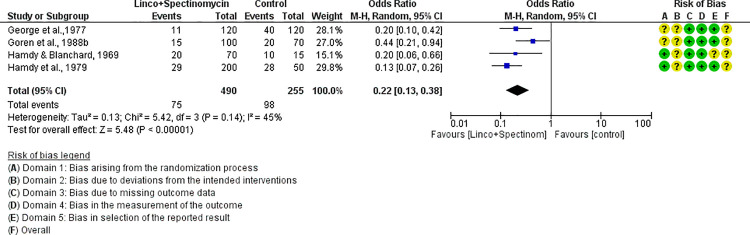
Forest plot of the efficacy of the combination of lincomycin *plus* spectinomycin to control mortality caused by colibacillosis in broilers.

**Fig 9:**
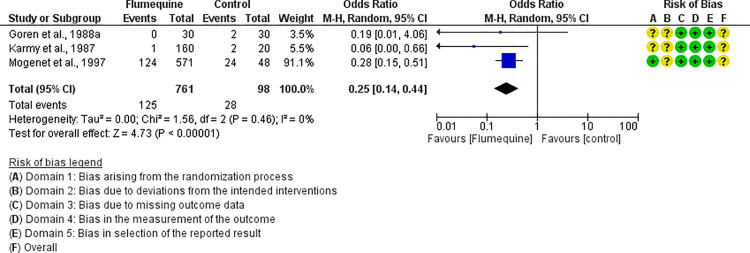
Forest plot of flumequine efficacy to control mortality caused by colibacillosis in broilers.

**Fig 10:**
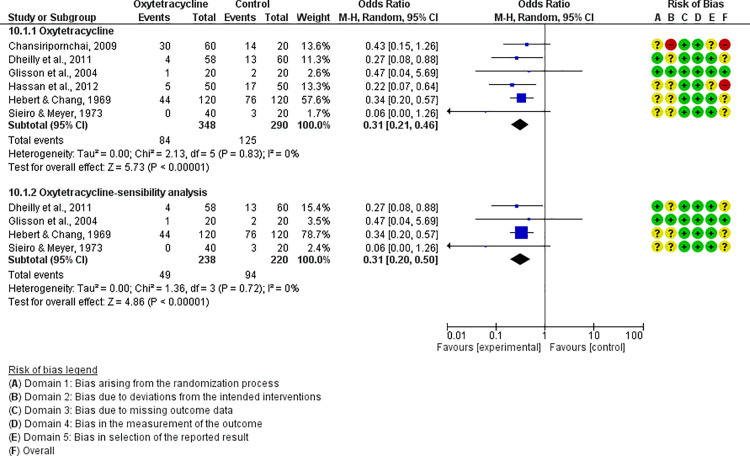
Forest plot of enrofloxacin efficacy to control mortality caused by colibacillosis in broilers.

**Fig 11:**
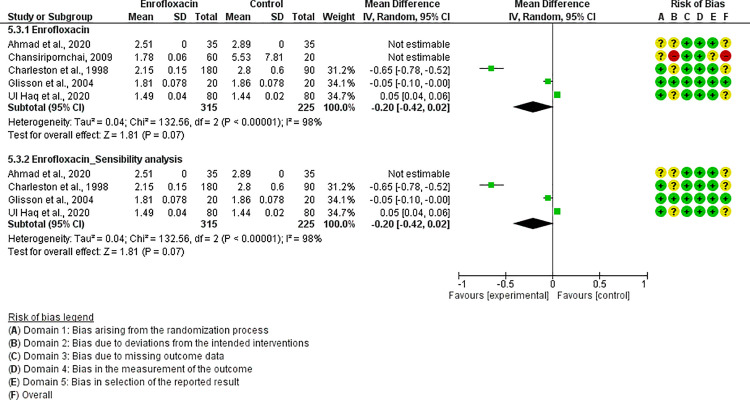
Forest plot of oxytetracycline effect considering the FCR assessment parameter.

For each antibiotic or combination of antibiotics with at least three studies, results were summarized either with an OR (for dichotomous outcomes, i.e., mortality) or mean difference (for continuous outcomes, i.e., FCR). The following antibiotics or combinations of antibiotics were subjected to pairwise meta-analysis: amoxicillin (mortality), spectinomycin (mortality), apramycin (mortality), neomycin (mortality), chlortetracycline (mortality), oxytetracycline (mortality and FCR), doxycycline (mortality and FCR), sulfadimethoxine (mortality and FCR), colistin (mortality), flumequine (mortality), enrofloxacin (mortality and FCR), and lincomycin *plus* spectinomycin (mortality). Doxycycline (Fig 5), enrofloxacin (Fig 6), spectinomycin (Fig 7), lincomycin *plus* spectinomycin (Fig 8) showed the strongest protective effect against mortality due to colibacillosis. Doxycycline (0.04; 95% CI 0.02-0.1; P < 0.001) recorded the highest protective effect followed by spectinomycin (0.11; 95% CI 0.05-0.26; P < 0.001), enrofloxacin (0.12; 95% CI 0.06-0.25; P < 0.00001) and lincomycin *plus* spectinomycin (0.22; 95% CI 0.13-0.38; P < 0.001). Flumequine (0.25; 95% CI 0.14-0.44; P < 0.001) and oxytetracycline (0.31; 95% CI 0.20-0.50; P < 0.001) showed moderate protective effects as presented in Figs 9 and 10, respectively. Regarding FCR, only enrofloxacin was considered in more than two studies; however, meta-analysis (Fig 11) showed a high heterogeneity across the studies.

### Risk of bias across studies

Publication bias was not performed due to the low number of studies included in the meta-analysis for each antibiotic or combination of antibiotics.

## Discussion

This systematic review aimed to summarize the evidence from the scientific literature regarding the efficacy of antibiotics in controlling colibacillosis in broiler production. A previous review by Sargeant et al. [28], which also focused on the efficacy of antibiotics to control colibacillosis in broilers naturally exposed to APEC, included only nine studies. The difference in the number of articles between the two reviews may be attributed to our choice to include RCTs that involved both natural and experimental exposure to APEC. Additionally, other methodological differences (e.g., search string, database interfaces, and libraries) may have contributed to the disparity in the inclusion of studies. In agreement with Sargeant et al. [28], we acknowledge that studies based on challenge trials might not be representative of the natural course of the disease under commercial husbandry conditions. Nevertheless, we included these studies to obtain data with a high certainty of evidence in accordance with the Grading of Recommendations Assessment, Development and Evaluation (GRADE) methodology [83]. This approach could be useful for developing evidence-based treatment guidelines for the European Network for Optimization of Antimicrobial Therapy (ENOVAT) guidelines (https://enovat.eu/).

Although the number of studies included in this review is relatively low, they cover a long period (earliest publications dating back to the 1960s) and a worldwide distribution (including several countries across four continents). An increasing trend in the number of articles published since the 2000s was also noted. These findings illustrate the enduring and growing interest in poultry colibacillosis as a serious disease affecting broilers, necessitating antimicrobial treatment to reduce mortality and economic losses in the poultry industry [3,84]. On the other hand, results from this review show incompleteness of reporting and extensive variation among the studies, including differences in the study design (e.g., settings, experimental units, number of animals per experimental group) and in the animal models (e.g., breed, sex, and age of birds), suggesting that more standardization across studies such as age of infection, type of stress to induce the disease, duration of experimentation is needed. Moreover, since the scientific literature in this review includes studies conducted over several years, potential bias might have been introduced due to the significant changes in modern broiler genetics and husbandry [85,86]. Out of 48 papers, only two investigated antibiotics as a prophylactic treatment [56,59] and four tested antibiotics as both prophylactic and metaphylactic treatments [37–39,43]. This finding suggests that efforts in primary research have been focused, particularly in recent years, on finding the most efficacious antibiotic to treat APEC infections in broilers. However, there is still a considerable lack of research that should comply with current policies to reduce the use of antibiotics in animals and to find alternatives to antibiotic treatment for poultry diseases.

The articles included in this review reported mortality (90%) as the most commonly measured outcome, followed by FCR (40%). Few studies provided information on condemnations at slaughter (4%), and total antibiotic use was never reported. The same outcomes were evaluated by Sargeant et al. [28], although that review found FCR to be the most frequently studied outcome. Our findings highlight that scientific research has predominantly focused on the antibiotic treatment of clinical disease, rather than on prophylactic use – a practice that should be abandoned in accordance with current policies aimed at banning or reducing the use of antibiotics as growth promoters. These policies, along with preventive measures, are essential for controlling infectious diseases in poultry production. Interestingly, more than half (n = 25; 52.1%) of the studies in this review included a score index for lesions as outcome that could be a suitable alternative or additional outcome for evaluating treatment efficacy in such studies. However, although most of the scoring systems were based on the severity of colibacillosis lesions (i.e., airsacculitis, pericarditis, and perihepatitis) observed during *post-mortem* examinations, there was a significant variability in how the scores were assigned to specific lesions [65,68]. Therefore, to ensure that lesion score is useful for comparison purposes, its use must be standardized across studies. Harmonizing lesions’ scoring systems would facilitate better comparisons and more accurate assessments of the efficacy of various treatments and interventions.

This review identified a wide variety of antibiotic interventions for treating colibacillosis in broiler production, encompassing antibiotics from several different classes (n = 18), and a wide variability for each administration method and treatment regime (e.g., dose and route of administration, frequency and duration of treatment). This wide diversity of antibiotics and treatment strategies tested in the studies likely reflects the ubiquitous and diverse nature of APEC and the extreme variability and severity of the clinical manifestations of colibacillosis, requiring specific antibiotic treatments [3,19,22]. Additionally, the increasing prevalence of resistance and multi-drug resistance of APEC over time [11,21,23], further complicates treatment strategies, necessitating the exploration of various antibiotics to effectively manage the disease. Given the current policy limiting the use of antibiotics in poultry production in the EU and globally [87,88], we conducted this systematic review with the aim to find scientific evidence for the efficacy of antibiotics to treat colibacillosis in broilers and suggest ideal antibiotics for therapy when necessary. The antibiotics tested in the studies span all the European Medicines Agency (EMA) categories (A = avoid; B = restrict; C = caution; and D = prudence) of antibiotics in the EU [89] (S3 Table). Notably, besides being listed in EMA category A (which use should be avoided), several antibiotics tested are currently not authorized for use in poultry in the EU due to restrictions [90,91] implemented over the years to ban or limit the use of antibiotics, for example, as growth promoters or due to their importance in human medicine [92]. However, it is important to note that the studies were conducted several years ago or in countries outside the EU, where these restrictions were or are not yet implemented. The most studied antibiotics (i.e., reported in ≥4 studies) were enrofloxacin (fluoroquinolone), oxytetracycline and doxycycline (tetracyclines), and spectinomycin (aminoglycoside) alone or associated with lincomycin (lincosamide). Enrofloxacin was the overall most studied antibiotic [50,51,56,57,61,63,67,72,73,75], likely due to its rapid and effective administration through water for treating acute respiratory diseases, including colibacillosis, in broilers [93,94]. Moreover, enrofloxacin has a broad-spectrum of activity not only against APEC but also against other important poultry respiratory pathogens, such as *Mycoplasma* spp. and *Pasteurella* spp. [26,93]. However, enrofloxacin is listed as a category B antibiotic (which use should be restricted) by EMA [89] and a Highest Priority Critically Important Antimicrobial (HPCIA) on the WHO list [92]. In contrast, tetracyclines and spectinomycin are classified as EMA category D (which use should be as first-line treatment), while lincosamides are in category C (which should be used when there are no alternatives in category D). Overall, these findings highlight the need for further research to evaluate the efficacy of antibiotics that can be used for colibacillosis treatment, addressing both the evolving situation regarding the prudent use of antibiotics and the safeguard of animal health and welfare. In addition, sharing results of trials performed by pharmaceutical companies and/or vertically integrated poultry operations could be crucial for bridging this gap.

A poultry-specific method [33] was used to assess the risk of bias in the studies included in this systematic review. This RoB method is based on the Cochrane Risk of Bias 2.0 tool [95], as adapted by Bueno et al. [33] to the specific poultry review question, similarly to our study. The overall RoB judgment for most studies was “some concerns” for both mortality and FCR outcomes. The primary area of bias was related to domain 2, which focuses on bias due to deviations from the intended interventions. Specifically, this was due to a lack of information regarding the researchers’ awareness of the broilers’ assigned interventions during the trial and potential deviations from the intended intervention that arose because of the trial context. Similar findings were observed in other reviews using the same poultry-specific RoB method [14,33]. Consequently, most studies in our review lacked to report key information of study design. As suggested by Moher et al. [34] and Sargeant et al. [96], poor reporting can be associated with exaggerated treatment effects. To address this issue, it is crucial to adopt a standardized method for reporting bias assessment in future studies to ensure complete information is easily accessible. Additionally, our findings suggest that protocols for such trials should be included, at least as supplementary materials, in publications, as this information could be essential for assessing the quality of the trial conducted and for explaining certain limitations in the results.

Due to the heterogeneity of the interventions and outcomes of the studies included in this systematic review, a pairwise meta-analysis was performed only for certain antibiotics (i.e., amoxicillin, spectinomycin, apramycin, neomycin, chlortetracycline, doxycycline, oxytetracycline, sulphadimethoxine, colistin, flumequine, enrofloxacin, and lincomycin *plus* spectinomycin combination) and for two outcomes (i.e., mortality and FCR). With few exceptions, these antibiotics are globally reported as commonly used in practice to treat colibacillosis in broilers [18–20]. Significant protective effects against mortality due to colibacillosis of spectinomycin, doxycycline, oxytetracycline, flumequine, enrofloxacin, and the lincomycin *plus* spectinomycin combination were detected by meta-analysis. Except for quinolones that are included in EMA category B, all the others belong to EMA categories C (i.e., lincosamides) and D (i.e., aminoglycosides, tetracyclines) [89]. Even though this is the first time a meta-analysis is performed to investigate the efficacy of antibiotics to control colibacillosis in broiler production, findings should be interpreted with caution due to the low number (<10) of studies included in the analysis, the variability of the animal model (e.g., age, breed), and the study design across studies. Additionally, meta-analysis did not consider parameters such as route of administration, dosage, frequency and duration of the treatment, that are crucial for a “judicious use” of antibiotics [93]. Variability in interventions (e.g., dose, timing of administration), outcome measurements (e.g., duration of the experiment, observation period), and other sources can significantly impact the different outcomes reported. To illustrate this issue, the sensitivity analysis for enrofloxacin considering mortality indicated higher heterogeneity when including the study by Chansiripornchai [61]. This study used a different broiler breed (i.e., Arbor Acres), a shorter study duration (7 days) and a different treatment frequency (twice a day). However, it cannot be established with certainty if these differences are the primary drivers of this heterogeneity, as the interplay between multiple factors cannot be discarded. Similarly, the removal of the study by Zolli & Polewaczyk et al. [38] during the sensitivity analysis significantly reduced the heterogeneity among the studies that assessed the efficacy of spectinomycin in controlling mortality caused by colibacillosis in broilers. This study was the only one where both metaphylactic and prophylactic treatments with spectinomycin were applied, and the duration of APEC inoculation was more than one day. Likewise, the evidence of the efficacy of oxytetracycline in controlling broiler mortality caused by colibacillosis was improved by removing the two studies [61,64] with high risk of bias from the meta-analysis. These results highlight the need for standardizing a broiler model to study the efficacy of antibiotic treatment. While our meta-analysis provides some evidence for the effectiveness of specific antibiotics in treating colibacillosis in broilers, the variability among studies underscores the necessity for standardized protocols. Future research should aim to harmonize study designs, intervention strategies, and outcome measures to produce more reliable and comparable data, ensuring that treatment recommendations are based on robust and consistent evidence.

## Conclusions

This systematic review aimed to quantitatively synthesize the evidence regarding the efficacy of antibiotics as an intervention for controlling colibacillosis in broiler production. Although we were able to conduct a meta-analysis for some antibiotics, we could not perform a network meta-analysis that would have allowed for the evaluation of the comparative efficacy of different treatment options. Consequently, based on our synthesis of the available literature, it is not possible to provide conclusive information regarding the most effective antibiotic option for colibacillosis treatment in broilers. However, we identified several research gaps – such as the need for standardization of animal models, harmonization of experimental conditions, adherence to current policies on antibiotic use in animals – that need to be urgently addressed. Addressing these gaps is crucial for refining treatment strategies for colibacillosis in broilers, in line with the evolving regulatory and public health guidelines.

It is advisable that future studies use a standard broiler model and are performed under similar experimental conditions. Additionally, they should focus on antibiotics from EMA category D or ultimately C. Conducting high-quality studies using less critical antibiotics within a standardized experimental model and conditions would facilitate the transition to more sustainable antimicrobial use in broilers and in the development of evidence-based guidelines for the treatment of colibacillosis, or other diseases, in broiler production.

## Supporting information

S1 Table Search string used to identify studies examining the efficacy of antibiotics to control colibacillosis in broiler production in four databases (CAB Abstracts, Agricola, Medline, and Web of Science). (DOCX)

S2 Table List of the papers analysed during the full text screening and the decisions made by reviewers. (DOCX)

S3 Table Classification of antibiotics tested in the studies according to the AMEG categorization and their authorization for use in Europe. (XLSX)

S4 Table PRISMA 2020 check list. (DOCX)
