# Supplementary material for: A systematic review and meta-analysis on the efficacy of antibiotic treatment in controlling colibacillosis in broiler production
Source: PLoS One. 2025 Jul 1;20(7):e0326535. doi: 10.1371/journal.pone.0326535 (PMC12212884; doi:10.1371/journal.pone.0326535)
Supplement: S1 Table — (DOCX) [file pone.0326535.s001.docx]

**S1 Table.** Search string used to identify studies examining the efficacy of antibiotics to control colibacillosis in broiler production in four databases (CAB Abstracts, Agricola, Medline, and Web of Science).

|  | **CAB Abstracts** | **Agricola** | **Medline** | **WOS** | **CAB Abstracts** | **Agricola** | **Medline** | **WOS** |
| --- | --- | --- | --- | --- | --- | --- | --- | --- |
|  | **First search**  **(March 2022)** | | | | **Second search**  **(November 2023)** | | | |
| #1 TS = (chicken* OR poultry* OR flock* OR gallus OR broiler*) | 238,904 | 111,591 | 145,423 | 491,633 | 241,637 | 117,853 | 157,236 | 520,555 |
| #2 TS = (antimicrobial* OR anti-microbial* OR antibiotic* OR antibacterial* OR anti-bacterial* OR apramycin OR amoxicillin OR avilamycin OR enrofloxacin OR neomycin OR neomicin OR salinomicyn OR salinomicin OR spectinomycin OR sulfaquinoxaline OR ceftiofur OR gentamycin OR gentamicin OR lincomycin OR oxytetracycline OR bacitracin OR sulfadimethoxine OR virginiamycin OR chlortetracycline OR tylosin OR tetracycline OR trimethoprim OR sulfamethoxazole OR penicillin OR flumequine OR ampicillin OR colistin OR ciprofloxacin) | 268,028 | 125,33 | 688,652 | 1,524,386 | 277,689 | 132,850 | 777,021 | 1,573,602 |
| #3 = TS (colibacillosis OR colisepticaemia OR peritonitis OR coli OR Escherichia OR coliform OR colisepticemia OR coligranuloma OR "Hjarre’s" OR "air sac disease" OR cellulitis OR osteomyelitis OR "brittle bone disease" OR salpingitis OR synovitis OR omphalitis OR enteritis OR "hemorrhagic septicemia" OR "chronic respiratory disease" OR "swollen head syndrome" OR "venereal colibacillosis" OR "coliform cellulitis" OR "yolk sac infection" OR APEC OR "pathogenic E. coli" OR “primary infection” OR “secondary infection” OR multifactorial OR multicausal) | 180,875 | 94,607 | 513,614 | 2,481,654 | 187,138 | 100,857 | 551,871 | 2,704,743 |
| #1 AND #2 AND #3 | 4,615 | 1,644 | 3,001 | 11,823 | 749 | 245 | 660 | 2,041 |
