# Supplementary material for: A systematic review and meta-analysis on the efficacy of antibiotic treatment in controlling colibacillosis in broiler production
Source: PLoS One. 2025 Jul 1;20(7):e0326535. doi: 10.1371/journal.pone.0326535 (PMC12212884; doi:10.1371/journal.pone.0326535)
Supplement: S2 Table — (DOCX) [file pone.0326535.s002.docx]

**S2 Table : List of the papers analysed during the full text screening and the decisions made by reviewers**

| **N^0^** | **References** | **Decisions of reviewers** |
| --- | --- | --- |
| 1 | Ahmed MH, Javed MT, Bahadur SU, Tariq A, Tahir MH, Tariq ME, Tariq N, Zarnab S, Ali MH. Antibacterial effects of copper oxide nanoparticles against E. coli induced infection in broilers. Applied nanoscience. 2022 Jul;12(7):2031-44 | Wrong outcome (no mortality or feed conversion ratio or condemnations at slaughter or total antibiotic use) |
| 2 | Mohamed Ahmed ZA, Mohamed EM, Abdelaziz Ali SG, Gergis AI. Assessment of meat protein quality of experimentally broilers fed diet supplemented with probiotics used as substitute to antibiotics in Luxor city. Assiut Veterinary Medical Journal. 2023 Jan 1;69(176):98-107. | Not related to colibacillosis |
| 3 | Meng J, Wang J, Zhu J, Li S, Qiu T, Wang W, Ding J, Wang W, Liu J. Bacteriostatic Effects of Yujin Powder and Its Components on Clinical Isolation of Multidrug-Resistant Avian Pathogenic Escherichia coli. Veterinary Sciences. 2023 May 4;10(5):328. | Not related to broilers |
| 4 | Abo Elfadl MA, Edress N, Abd Elalim AE, Arafa MM. Biomarkers of antioxidant and biochemical for broiler chicks infected with e. Coli treated with probiotiC. Biochemical & Cellular Archives. 2022 Apr 1;22(1). | Not related to colibacillosis |
| 5 | Sarfraz M, Nguyen TT, Wheler C, Köster W, Gerdts V, Dar A. Characterization of dosage levels for in ovo administration of innate immune stimulants for prevention of yolk sac infection in chicks. Veterinary Sciences. 2022 Apr 22;9(5):203. | Not related to antibiotics |
| 6 | Khaliq, Kashfa; Mudassar, Muhammad; Rasool, Muhammad Hidayat; Aslam, Muhammad Aamir; Zafar, Nishat; Khatoon, Aisha; Ali, Ashiq; Ashraf, Muhammad Umar; Iqbal, Javed; Raza, Ali; Determine the therapeutic and immunomodulatory effect of Aloe vera polysaccharides against colibacillosis in chickens. Bioscience Research, 2022, 19(1):60-67. | Not related to antibiotics |
| 7 | Gu Y, Cao Y, Wang X, Shan C, Liu Y, Jiang G. Effect of Fermented Traditional Chinese Medicine on Intestinal Microflora of Broilers with Avian Colibacillosis. Journal of Henan Agricultural Sciences. 2022 Jul 15;51(7):145. | Not english, french or spanish |
| 8 | Helmy YA, Kathayat D, Deblais L, Srivastava V, Closs Jr G, Tokarski RJ, Ayinde O, Fuchs JR, Rajashekara G. Evaluation of novel quorum sensing inhibitors targeting auto-inducer 2 (AI-2) for the control of avian pathogenic Escherichia coli infections in chickens. Microbiology Spectrum. 2022 Jun 29;10(3):e00286-22. | Not related to antibiotics |
| 9 | Merzlenko, O. V., Noskov, S. B., Gorbach, A. A., & Pozdnyakova, V. N. (2022). The efficiency of a combined antibiotic for the therapy of mixed bacterial infections in broilers. | Not english, french or spanish (Russian) |
| 10 | Sedov, S. A., Shchukin, K. I., Dorofeeva, S. G., Anosov, D. E., & Khoshafyan, L. S. (2022). The efficiency of the combination of a customized antibacterial drug and a vaccine against colibacillosis in commercial broiler production. | Not english, french or spanish |
| 11 | Betts JW, Cawthraw S, Smyth JA, Poole RK, Roth P, Schatzschneider U, La Ragione RM. The manganese carbonyl complex [Mn (CO) 3 (tqa-κ3N)] Br: A novel antimicrobial agent with the potential to treat avian pathogenic Escherichia coli (APEC) infections. Veterinary microbiology. 2023 Sep 1;284:109819. | Not related to antibiotics and wrong outcome (no mortality or feed conversion ratio or condemnations at slaughter or total antibiotic use) |
| 12 | Jhandai P, Mittal D, Gupta R, Kumar M, Khurana R. Therapeutics and prophylactic efficacy of novel lytic Escherichia phage vB_EcoS_PJ16 against multidrug-resistant avian pathogenic E. coli using in vivo study. International Microbiology. 2024 Jun;27(3):673-87.  pathogenic E. coli using in vivo study. | Not related to colibacillosis |
| 13 | Umesha, B. U., G. Ananda Manegar, and B. E. Shambulingappa. "Efficacy of apramycin in prevention and treatment of E. coli infection in broilers." (2010): 824-827. | Full text not available |
| 14 | Akbar H, Khan M, Khan AA, Khan MA, Shuaib M, Akbar SF, Manzoor S, Irshad-ur-rehman SA, Ali L, Khalid R, Idrees M. Comparative efficacy of doxycycline and flumequine against experimentally induced colibacillosis in broiler chicks. Journal of Veterinary Medicine and Animal Health. 2009 Aug;1(2):017-22. | Duplicate |
| 15 | Ezhov, V. I. "Effectiveness of antibiotics in experimental coli septicaemia in chicks (particularly tetracyclines, chloramphenicol, streptomycin and kanamycin)." (1975): 63-66. | Not english, french or spanish (Russian) |
| 16 | Teo AL, Tan HM. Effect of Bacillus subtilis PB6 (CloSTAT) on broilers infected with a pathogenic strain of Escherichia coli. Journal of applied poultry research. 2006 Jul 1;15(2):229-35. | Not related to colibacillosis and not related to antibiotics |
| 17 | Ullah A, Anjum AA, Rabbani M, Ijaz M, Nawaz M, Ashraf M, Ali A, Rashid A, Najeeb I, Hussain A. Activity of ethanolic extract of Eucalyptus globulus leaves against multi drug resistant poultry pathogens in broiler chicks. Cellular and Molecular Biology. 2021 Jan 31;67(1):153-8. | Not related to antibiotics and wrong outcome (no mortality or feed conversion ratio or condemnations at slaughter or total antibiotic use) |
| 18 | Mazur AD, Yurin DV, Skvortsov VN, Tarasova YV. Acute toxicity and comparative therapeutic efficacy of levofloxacin in experimental colibacillosis of chickens. International Journal of Veterinary Medicine. 2020(3):89-93. | Not english, french or spanish |
| 19 | Handharyani E, Sutardi LN, Mustika AA, Andriani A, Yuliani S. Antibacterial activity of Curcuma longa (turmeric), Curcuma zedoaria (zedoary), and Allium sativum (garlic) nanoparticle extract on chicken with chronic respiratory disease complex: in vivo study. InE3S web of conferences 2020 (Vol. 151, p. 01054). EDP Sciences. | Not related to colibacillosis |
| 20 | Geidam YA, Ambali AG, Onyeyili PA, Tijjani MB, Gambo HI, Gulani IA. Antibacterial efficacy of ethyl acetate fraction of Psidium guajava leaf aqueous extract on experimental Escherichia coli (O78) infection in chickens. Veterinary World. 2015 Mar;8(3):358. | Wrong outcome (no mortality or feed conversion ratio or condemnations at slaughter or total antibiotic use) |
| 21 | Baliarsing, S. K., A. G. Rao, P. R. Mishra, and B. N. Mohanty. "Antibiotic sensitivity test against colibacillosis in broiler chicks." (1991): 142-143. | Full text not available |
| 22 | Ghosh TK, Haldar S, Bedford MR, Muthusami N, Samanta I. Assessment of yeast cell wall as replacements for antibiotic growth promoters in broiler diets: effects on performance, intestinal histo‐morphology and humoral immune responses. Journal of Animal Physiology and Animal Nutrition. 2012 Apr; 96(2):275-84. | Not related to antibiotics |
| 23 | Otarov AI. Bactericidal action of antimicrobial preparations on pathogenic Escherichia isolated from hens. Veterinariia. 1980 Jan(1):66-7. | Not english, french or spanish |
| 24 | Sugiharto, Sugiharto, T. Yudiarti, I. Isroli, E. Widiastuti, H. I. Wahyuni, T. A. Sartono, A. N. Al-Baarri, and N. Nurwantoro. "Breast muscle characteristics of avian pathogenic Escherichia coli infected broilers fed with antibiotics or probiotic." (2019): 131-140. | Wrong outcome (no mortality or feed conversion ratio or condemnations at slaughter or total antibiotic use) |
| 25 | Trailović, D., V. Nikolić, B. Marković, V. Panajotović, and Z. Jurišić. "Ceftriaxone and its use in veterinary medicine." (1992): 7-12. | Not english, french or spanish (Serbo-croatian) |
| 26 | Zaikina EN, Skvortzov VN, Balbutskaya AA. Ciprofloxacin efficacy in an experimental colibacteriosis of chickens. International Bulletin of Veterinary Medicine. 2015. | Full text not available |
| 27 | Raheel, I. A. R., A. Orabi, S. Hassan, and A. El-Masry. "Cleanactiv® combatting crisis of multidrug resistant avian pathogenic E. coli in broiler chickens." (2019): 283-288. | Wrong outcome (no mortality or feed conversion ratio or condemnations at slaughter or total antibiotic use) |
| 28 | Roliński, Z., C. Kowalski, P. Właź, and R. Furmaga. "Combined activity of colistin and tylosin against pathogenic bacteria." (1996): 242-245. | Not english, french or spanish (Polish) |
| 29 | Prukner, E.; Vukovic, B.; Ragus-ETHuric, R.; Milakovic-Novak, L.; Nemanic, J.Comparative studies on the in-vitro and in-vivo activity of some antibiotics and the results of their use in poultry production. Usporedna istrazivanja in vitro i in vivo aktivnosti nekih antibiotika i uspjeh njihove primjene u peradarskoj proizvodnji. - Volume 41, Issue 1, pp. 33-41, 1987-01-01 | Not english, french or spanish (but in Serbo-Croatian) |
| 30 | Kakooza, S., W. Eneku, D. Ayebare, D. Ndoboli, I. Mbatidde, J. Waiswa, M. Barasa, Kristina Roesel, and Arshnee Moodley. "Training manual for frontline animal extension service providers on antimicrobial resistance in poultry production." (2023). | Full text not available |
| 31 | Sun HuaiGang, Sun HuaiGang, Wang Xin Wang Xin, Cui YiZhe Cui YiZhe, and Shao Hong Shao Hong. "Curative effect experiment of synergistic antibacterial Chinese herb compound on artificial infected chicken colibacillosis." (2011): 86-89. | Not english, french or spanish (but in Chinese) |
| 32 | Yin ShuTian, Yin ShuTian, and Su JianHua Su JianHua. "Dosage screening tests of norfloxacin, chloromycetin and vibramycin for treatment of colibacillosis in broilers." (1997): 15-16. | Not english, french or spanish (but in Chinese) |
| 33 | Ezhov VI, Piskunov GM, Krylov NA, Bessonov IG, Boev IS, Burlakova RN, Golumenov MG, Shkarin NV. Effectiveness of antibiotics for respiratory mycoplasmosis and colisepticaemia of poultry. Biulleten'Vsesoiuznogo instituta eksperimental'noi veterinarii. 1978. | Full text not available |
| 34 | Ezhov VI, Piskunov GM, Krylov NA, Bessonov IG, Boev IS, Burlakova RN, Golumenov MG, Shkarin NV. Effectiveness of antibiotics for the control of mycoplasmosis and septicemia caused by Escherichia coli in poultry. Veterinariia Mosk. 1977. | Not english, french or spanish |
| 35 | Ezhov VI. Effectiveness of antibiotics in experimental colisepticemia in chicks. Veterinariia. 1975 Sep 1(9):63-6. | Not english, french or spanish |
| 36 | Bessarabov BF, D'iakonova EV, Rodin IV, Motylev VF, Shumilova IN. Effectiveness of spectam in chicken coliform septicemia. Veterinariia. 1975 Mar(3):58-60. | Not english, french or spanish |
| 37 | Artem'eva SA, Babaeva MV, Andriianov NS. Effectiveness of treatment of Escherichia coli infection of the chickens. Veterinariia. 1977 Sep 1(9):62-3. | Not english, french or spanish |
| 38 | H. SUMANO L, Ocampo CL, Brumbaugh GW, Lizarraga RE. Effectiveness of two fluoroquinolones for the treatment of chronic respiratory disease outbreak in broilers. British poultry science. 1998 Mar 1;39(1):42-6. | Not related to colibacillosis |
| 39 | Ezhov VI, Grosheva GA, Minaev IM. Effect of antibacterial preparations on the causative agent of avian colisepticemia. Veterinariia. 1970 Dec;12:33-4. | Not english, french or spanish |
| 40 | Isroli I, Yudiarti T, Widiastuti E, Wahyuni HI, Sartono TA, Sugiharto S. Effect of Bacillus probiotics on internal organs and carcass characteristics of broiler chicks infected with avian pathogenic Escherichia coli. Livest. Res. Rural Dev. 2018;30(11). | Wrong outcome (no mortality or feed conversion ratio or condemnations at slaughter or total antibiotic use) |
| 41 | Amerah AM, van Rensburg CJ, Plumstead PW, Kromm C, Dunham S. Effect of feeding diets containing a probiotic or antibiotic on broiler performance, intestinal mucosa-associated avian pathogenic E. coli and litter water-soluble phosphorus. Journal of Applied Animal Nutrition. 2012 Jan;1:e7. | Not related to antibiotics |
| 42 | Kincannon, R. W., D. E. Polewaczyk, and Z, Jr Zolli. "Effect of spectinomycin alone or in combination with lincomycin on experimentally induced airsacculitis associated with Mycoplasma gallisepticum or Escherichia coli in chickens." (1974): 1944. | Full text not available |
| 43 | Viaene, N., and A. Devos. "Effect of spectinomycin on experimental infection of air sacs in broilers with Escherichia coli." (1973): 114-119. | Not english, french or spanish (Dutch) |
| 44 | Abiodun BS, Adedeji AS, Taiwo O, Gbenga A. Effects of Moringa oleifera root extract on the performance and serum biochemistry of Escherichia coli challenged broiler chicks. Journal of Agricultural Sciences, Belgrade. 2015;60(4):505-13. | Not related to colibacillosis |
| 45 | Kubasa, P. "Efficacy of Apralan (apramycin) in the control of E. coli infection in poultry." (1988): 91-95. | Not english, french or spanish (Polish) |
| 46 | Mazurkiewicz, M., A. Latała, A. Wieliczko, A. Zalesiński, and M. Tomaszewski. "Efficacy of Baytril in the control of bacterial diseases of poultry." (1990): 286-289. | Not english, french or spanish (Polish) |
| 47 | Fang BingHu, Fang BingHu, Zeng ZhenLing Zeng ZhenLing, Feng QiHui Feng QiHui, and Chen ZhangLiu Chen ZhangLiu. "Efficacy of enrofloxacin against experimentally induced colibacillosis and staphylococcosis in chickens." (1997): 157-160. | Not english, french or spanish (Chinese) |
| 48 | Li XiaoJun, Li XiaoJun, Du AiFang Du AiFang, Ma YouZhi Ma YouZhi, and Yu DeTai Yu DeTai. "Efficacy of flumequine soluble powder against experimental Escherichia coli infection in chickens." (2004): 9-10. | Not english, french or spanish (Chinese) |
| 49 | Fernandez, A., Lara, C., Puyuelo, R., Gomez, J., Ramos, J.J., Loste, A., Marca, M.C. and Verde, M.T., 1998. Efficacy of phosphomycin in the control of Escherichia coli infection of broiler chickens. *Research in veterinary science*, *65*(3), pp.201-204. | Wrong outcome (no mortality or feed conversion ratio or condemnations at slaughter or total antibiotic use) |
| 50 | Lu YongSiu, Lu YongSiu, Tsai HsiangTsai Tsai HsiangTsai, and Sung TwaTsung Sung TwaTsung. "Efficacy of quinolones on experimental avian mycoplasma infections and/or colibacillosis in young chickens." (1995): 21-26. | Not english, french or spanish (Chinese) |
| 51 | Roliński, Z., M. Duda, A. Gaweł, C. Kowalski, and P. Wlaź. "Efficacy of tylosin and colistin combination in the treatment of bacterial infections of animals." (1996): 440-442. | Not english, french or spanish (Polish) |
| 52 | Crivineanu, M., V. Crivineanu, and G. V. Goran. "Enrofloxacin's efficacy in some birds bacterial diseases studies." (2002): 129-136. | Not english, french or spanish (Romanian) |
| 53 | White G, Williams RB. Evaluation of a mixture of trimethoprim and sulphaquinoxaline for the treatment of bacterial and coccidial diseases of poultry. The Veterinary Record. 1983 Dec 1;113(26-27):608-12. | Not english, french or spanish |
| 54 | Galal HM, Abdrabou MI, Faraag AH, Mah CK, Tawfek AM. Evaluation of commercially available aroA delated gene E. coli O78 vaccine in commercial broiler chickens under Middle East simulating field conditions. Scientific Reports. 2021 Jan 21;11(1):1938. | Not related to antibiotics (concerning vaccination) |
| 55 | Giebel, O., M. Mazurkiewicz, A. Mróz, T. Pietrzkiewicz, and A. Zalesiński. "Evaluation of the efficacy of Apralan (apramycin) in controlling bacterial diseases of poultry." (1984): 137-140. | Not english, french or spanish (Polish) |
| 56 | Timms LM, Marshall RN, Breslin MF. Evaluation of the efficacy of chlortetracycline for the control of chronic respiratory disease caused by Escherichia coli and Mycoplasma gallisepticum. Research in veterinary science. 1989 Nov 1;47(3):377-82. | Not related to colibacillosis |
| 57 | Giebel, O., M. Mazurkiewicz, A. Mróz, T. Pietrzkiewicz, and A. Wieliczko. "Evaluation of the efficacy of Imequyl (flumequine) in the control of bacterial diseases of poultry." (1984): 106-109. | Not english, french or spanish (Polish) |
| 58 | Donos, A. "Experimental use of Apramycin against broiler colibacillosis." (1982): 65-70. | Not english, french or spanish (Greek) |
| 59 | Donos, A. "Experimental use of apramycin against broiler colibacillosis [Chickens, Greece]." *Deltion= Bulletin of the Hellenic Veterinary Medical Society* (1982). | Not english, french or spanish (Greek) and duplicate |
| 60 | Long T; Florfenicol injection composition useful for treating diseases of livestock and poultry e.g. fowl Colibacillosis, Salmonellosis, Pasteurella disease comprises florfenicol, organic solvent, complexing agent and antioxidant in specific amount | Full text not available |
| 61 | Wahjuni RS, Sabdoningrum EK, Hidanah S. Immunomodulation Effect of Meniran (Phyllanthus Niruri Linn) on blood profile of Broiler Chickens with enterotoxin of antibiotic resistant Escherichia coli. InProceeding The 1st International Conference Postgraduate School. Advances in Social Science, Education and Humanities Research (Assehr) 2017 (Vol. 98, pp. 311-313). Atlantis Press. | Wrong outcome (no mortality or feed conversion ratio or condemnations at slaughter or total antibiotic use) |
| 62 | Anonymous. Individual and combined efficacy of bacteriophage and Baytril (R) (enrofloxacin) to treat a severe Escherichia coli respiratory infection in broiler chickens. POULTRY SCIENCE - Volume 83, Issue 10, pp. 1780-1781 - published 2004-01-01 | Full text not available |
| 63 | Ogunbanwo, S. T., A. I. Sanni, and A. A. Onilude. "Influence of bacteriocin in the control of Escherichia coli infection of broiler chickens in Nigeria." *World Journal of Microbiology and Biotechnology* 20 (2004): 51-56. | Not related to antibiotics |
| 64 | Śmiałek M, Kowalczyk J, Koncicki A. Influence of vaccination of broiler chickens against Escherichia coli with live attenuated vaccine on general properties of E. coli population, IBV vaccination efficiency, and production parameters—A field experiment. Poultry science. 2020 Nov 1;99(11):5452-60. | Not related to antibiotics (concerning vaccination) |
| 65 | Guo HaiYong, Guo HaiYong, Yuan HongXing Yuan HongXing, Wang YunXiao Wang YunXiao, Xu RuiTao Xu RuiTao, and Song QinYe Song QinYe. "Inhibitory effects of recombinant porcine beta-defensin 1 on Escherichia coli." (2017): 1342-1348. | Not english, french or spanish (Chinese) |
| 66 | Spiler, E., D. Josipovic, and B. Gliha. "Nifurprazine in the treatment of Escherichia coli infection in fowls." (1981): 283-288. | Not english, french or spanish (Serbo-croatian) |
| 67 | Kathayat D, Helmy YA, Deblais L, Srivastava V, Closs Jr G, Khupse R, Rajashekara G. Novel small molecule growth inhibitor affecting bacterial outer membrane reduces extraintestinal pathogenic Escherichia coli (ExPEC) infection in avian model. Microbiology Spectrum. 2021 Oct 31;9(2):e00006-21. | Not use of a comparator group during the trial |
| 68 | Dmitrieva, M. E., O. B. Novikova, and L. M. Kashkovskaya. "Overcoming resistance in treatment of colibacteriosis in poultry." (2017): 33-37. | Not english, french or spanish (Russian) |
| 69 | Xiao, X. L., J. Z. Shen, B. L. Zhu, S. G. Liu, Z. X. Wang, and Y. C. Dong. "Pharmacodynamical study of Tylo-Tad plus and furaltadone in colibacillosis and salmonellosis." (1993): 81-86. | Not english, french or spanish (Chinese) |
| 70 | Abo El-Ela FI, Radi AM, El-Banna HA, El-Gendy AA, Tohamy MA. Pharmacokinetics of difloxacin in healthy and E. coli-infected broiler chickens. British poultry science. 2014 Nov 2;55(6):830-6. | Wrong outcome (no mortality or feed conversion ratio or condemnations at slaughter or total antibiotic use) |
| 71 | Ziv G. Preliminary clinical pharmacological investigations of tylosin and tiamulin in chickens. Veterinary Quarterly. 1980 Oct 1;2(4):206-10. | Not broilers and not related to colibacillosis |
| 72 | Wideman Jr RF, Al-Rubaye A, Kwon YM, Blankenship J, Lester H, Mitchell KN, Pevzner IY, Lohrmann T, Schleifer J. Prophylactic administration of a combined prebiotic and probiotic, or therapeutic administration of enrofloxacin, to reduce the incidence of bacterial chondronecrosis with osteomyelitis in broilers. Poultry Science. 2015 Jan 1;94(1):25-36. | Not related to colibacillosis |
| 73 | Labarthe, JC; Guillot, Jf; Mouline, C; Bree, A. Quantitative study of antibacterial activity of cefotaxime and ceftriaxone during experimental escherichia-coli k1 bacteremia in chickens. Pathologie Biologie - Volume 37, Issue 5, pp. 664-667 - published 1989-01-01 | Duplicate |
| 74 | Cherneva, E., and L. Karaivanov. "Response to some antibiotics of streptomycin-dependent and streptomycin-resistant Escherichia coli mutants, pathogenic to birds [Chickens]." *Veterinarno-meditsinski nauki.= Veterinary science* (1981). | Not english, french or spanish |
| 75 | Druz’ EA, Kashnikova TV, Khmyrov AV, Fel’dman NB, Lutsenko SV. Safety and efficacy of a new antimicrobial liposomal drug in raising broiler chicks. Russian Agricultural Sciences. 2009 Jun;35:202-4. | Not related to colibacillosis |
| 76 | Mazurkiewicz, M., O. Giebel, A. Wieliczko, A. Zalesiński, and A. Galantowicz. "Studies on the efficacy of Paracilline in the control of bacterial diseases in poultry." (1990): 376-379. | Not english, french or spanish (Polish) |
| 77 | Koncicki, A., A. Krasnodębska-Depta, W. Szweda, M. Andrzejewski, and J. Olkowski. "Studies on the usefulness of norfloxacin in the therapy of bacterial diseases in poultry." (2002): 52-55. | Not english, french or spanish |
| 78 | Zolli, Z., and D. E. Polewaczyk. "Studies on the use of spectinomycin to control several diseases of poultry: chronic respiratory disease (Escherichia coli), Salmonella typhimurium, Arizona Group infection and fowl cholera." *World Vet Poultry Ass Int Congr Pap* (1970). | Not english, french or spanish |
| 79 | Cristina RT, Schmerold I, Dumitrescu E, Lăzărescu C, Petrovici S, Netotea A. Study of Doxicycline efficacy in broilers’ Colibacilosis. Scientific Papers Animal Science and Biotechnologies. 2010;43(1):469 | No original research |
| 80 | Zolli, E. "Study of the use of spectinomycin in the control of some diseases in poultry.(Chronic respiratory disease (Escherichia coli), Salmonella typhymurium, Arizona group of infections and fowl cholera)." *Veterinarski glasnik* (1970). | Duplicate |
| 81 | Wientarsih, Ietje, Sus Derthi Widhyari, and Tika Aryanti. "The combination of curcumin with zinc in feed as alternatif therapy collibaciilosis in broiler." (2013): 327-334. | Not english, french or spanish (Indonosian) |
| 82 | Gorbach, A. A., V. N. Pozdnyakova, and S. B. Noskov. "The comparative study of the efficiency of preparation" Floxagen S" against a bacterial infection in broilers." (2020): 45-49. | Not english, french or spanish (Russian) |
| 83 | Gorbach, A. A., V. N. Pozdnyakova, and S. B. Noskov. "The comparative study of the efficiency of preparation" Floxagen S" against a bacterial infection in broilers." (2020): 45-49. | Duplicate |
| 84 | Ezhov, V. I., G. A. Grosheva, and I. M. Minaev. "effect of antibacterial drugs on the pathogen of coli septicemia in poultry." *Veterinariia* (1970). | Not english, french or spanish |
| 85 | Leitner G, Waiman R, Heller ED. Pharmacology: The effect of apramycin on colonization of pathogenic Escherichia coli in the intestinal tract of chicks. Veterinary Quarterly. 2001 Apr 1;23(2):62-6. | Wrong outcome (no mortality or feed conversion ratio or condemnations at slaughter or total antibiotic use) |
| 86 | Fitri AN, Fitriana I, Rosetyadewi AW, Pratama AM, Septiana AI, Setiawan DC, Wijayanti AD. The effect of colistin administration as medicated feed on alanine aminotransferase and creatinine level in broiler infected with Escherichia coli. InBIO Web of Conferences 2021 (Vol. 33, p. 03002). EDP Sciences. | Wrong outcome (no mortality or feed conversion ratio or condemnations at slaughter or total antibiotic use) |
| 87 | Prisnyi, A. A., A. A. Moiseeva, and V. N. Skvortsov. "The effect of cyprofloxacin on the leukogramm of chickens blood with experimental colibacillosis." (2019): 28-32. | Not english, french or spanish (Russian) |
| 88 | Viaene, N.; Devos, A.; The effect of spectinomycin on experimental Escherichia coli air sac infection in broiler chickens. De invloed van Spectinomycine op experimentele E. coli infecties van de luchtzakken bij mestkuikens - Volume 42, Issue 3, pp. 114-119 - published 1973-01-01 | Duplicate |
| 89 | Glisson JR. The efficacy of enrofloxacin (Baytril) for the treatment of colibacillosis in chickens and turkeys and fowl cholera in turkeys. InWestern Poultry Disease Conference (USA) 1996. | Full text not available |
| 90 | Jiang YW, Sims MD, Conway DP. The efficacy of TAMUS 2032 in preventing a natural outbreak of colibacillosis in broiler chickens in floor pens. Poultry science. 2005 ;84(12):1857-9. | Not related to colibacillosis |
| 91 | Merzlenko, Oksana V., Sergey B. Noskov, Alexander A. Gorbach, and Valentina N. Pozdnyakova. "The efficiency of a combined antibiotic for the therapy of mixed bacterial infections in broilers." (2022): 65-68. | Duplicate |
| 92 | Dorozhkin, V. I., A. A. Gorbach, and A. A. Reznichenko. "The efficiency of alternatives to antibiotics in the therapy of colibacteriosis in broilers." (2020): 70-74. | Not english, french or spanish |
| 93 | Vangelov S, Iovchev E, Simov I, Mentov V. Therapeutic effect and residues of a Gentavet preparation in chicks, piglets and calves. Veterinarno-meditsinski Nauki. 1986 Jan 1;23(1):3-9. | Not english, french or spanish |
| 94 | Hamdy AH. Therapeutic effect of Linco-Spectin on airsacculitis in chickens. Avian diseases. 1970 Nov 1:706-14. | Not related to colibacillosis |
| 95 | Bao J, Zhang Y, Zhang L, Gong X, Shi W, Liu L, Wang X. Therapeutic effect of Schisandrin A on avian colibacillosis through gut-liver axis. Poultry Science. 2021 Oct 1;100(10):101371. | Wrong outcome (no mortality or feed conversion ratio or condemnations at slaughter or total antibiotic use) |
| 96 | Babkova, E.A.; Yunyaev, N.V.; Therapy of colibacteriosis in broiler chickens in modern industrial poultry farming. Veterinariya - Volume 0, Issue 9, pp. 23-25 - published 2017-01-01 | Full text not available |
| 97 | Sasipreeyajan, Jiroj, and Somsak Pakpinyo. "The result of using enrofloxacin in broiler chickens after experimental E. coli infection." (1992): 95-104. | Not english, french or spanish (Thai) |
| 98 | Liang HuaLi, Liang HuaLi, Xu Hui Xu Hui, Hua JiongGang Hua JiongGang, Gu YaXian Gu YaXian, Yang GuanMao Yang GuanMao, and Wu WanFu Wu WanFu. "The therapeutic trial of soluble powder of enrofloxacin sodium on chickens experimentally infected with E. coli." (1998): 313-315. | Not english, french or spanish (Chinese) |
| 99 | Youssef, Sah; Atef, M; Elbanna, HA; Hanafy, MSM; Abonorge, M; Elkatan, YA; Haagsma, N; Ruiter, A; Czedikeysenberg, PB. Tissue residue depletion of sulfadimethoxine in healthy and escherichia-coli infected broiler-chickens. Residues of veterinary drugs in food, vols 1 and 2, pp. 696-700 - published 1993-01-01 | Not related to colibacillosis and not related to antibiotics (concerning antibiotic residues) |
| 100 | Anonymous. Treatment of experimental avian colibacteriosis | Full text not available |
| 101 | Kromann S, Kudirkiene E, Li L, Thoefner I, Daldorph E, Christensen JP, Meng H, Olsen RH. Treatment with high-dose antidepressants severely exacerbates the pathological outcome of experimental Escherichia coli infections in poultry. PLoS One. 2017 Oct 11;12(10):e0185914. | Not broiler and not related to colibacillosis |
| 102 | Boratto AJ, Lopes DC, Oliveira RF, Albino LF, Sá LM, Oliveira GA. Use of antibiotic, probiotic and homeopathy, inoculated or not with Escherichia coli, for broilers reared under comfort environment. Revista Brasileira de Zootecnia. 2004;33:1477-85. | Wrong outcome (no mortality or feed conversion ratio or condemnations at slaughter or total antibiotic use) |
| 103 | Schmittle, S. C. "Use of dibenzylethyl-enediamine dipenicillin G and dihydrostrepto-mycin sulfate in chronic respiratory disease of chickens." (1954): 221-223. | Not related to colibacillosis |
| 104 | Gautrais B, Copeland D. Use of enrofloxacin against colibacillosis in chickens. InWestern Poultry Disease Conference (USA) 1997. | Full text not available |
| 105 | Cracknell VC, Andreotis J, Facibeni G, Owais E, Pradella G. An evaluation of apramycin soluble powder for the treatment of naturally acquired Escherichia coli infections in broilers. J. Vet. Pharmacol. Ther. 1986;9(3):273-279. | Include |
| 106 | Hamdy AH, Blanchard CJ. Effect of lincomycin and spectinomycin water medication on chickens experimentally infected with Mycoplasma gallisepticum and Escherichia coli. Poult. Sci. 1969;48(5):1703-1708. doi: 10.3382/ps.0481703 | Include |
| 107 | Hebert TJ, Chang TS. The effect of furazolidone and other drug on artificially induced Escherichia coli infection in chicken. Poult. Sci. 1969;48(6):2063-2069. doi: 10.3382/ps.0482063 | Include |
| 108 | Zolli Z, Polewaczyk DE. The use of spectinomycin to control experimentally induced airsacculitis associated with Escherichia coli in chickens and turkeys. Avian Dis. 1971;15(3): 477-482. doi: 10.2307/1588724 | Include |
| 109 | Sieiro F, Meier FA. Activity of sulphachloropyridazine against experimentally induced E. coli infection in broiler chickens. Vet. Rec. 1973;49(12):303-310. | Include |
| 110 | George BA, Fagerberg DJ, Quarles CL, Fenton JM. Comparison of therapeutic efficacy of doxycycline, chlortetracycline and lincomycin-spectinomycin on E. coli infection of young chickens. Poult. Sci. 1977;56(2):452-458. doi: 10.3382/ps.0560452 | Include |
| 111 | Hamdy AH, Kratzer DD, Paxton LM, Roberts BJ. Effect of a single injection of lincomycin, spectinomycin, and lincospectin on early chick mortality caused by Escherichia coli and Staphylococcus aureus. Avian Dis. 1979;23(1):164-173. doi: 10.2307/1589683 | Include |
| 112 | Goren E, de Jong WA, Doornenbal P. Some pharmacokinetic aspects of four sulphonamides and trimethoprim, and their therapeutic efficacy in experimental Escherichia coli infection in poultry. Vet. Q. 1984;6(3):134-140. doi: 10.1080/01652176.1984.9693927 | Include |
| 113 | Karmy SA, Shouman MT, Ragab AM, Safwat EEA, El-Danaf N. Studies on the efficacy and levels of flumequine in healthy and experimentally infected birds with E. coli 078:K80 in vitro and in vivo. J. Egypt. Vet. Med. Ass. 1987;47(1/2):521-536. | Include |
| 114 | Goren E, de Jong WA, Doornenbal P. Additional studies on the therapeutic efficacy of sulphadimidine sodium in experimental Escherichia coli infection of broilers. Vet. Q. 1987;9(1):86-87. doi: 10.1080/01652176.1987.9694081 | Include |
| 115 | Goren E, de Jong WA, Doornenbal P, Laurense T. Therapeutic efficacy of doxycycline hyclate in experimental Escherichia coli infection in broilers. Vet. Q. 1988;10(1):48-52. doi: 10.1080/01652176.1988.9694145 | Include |
| 116 | Goren E, de Jong WA, Doornenbal P. Therapeutic efficacy of medicating drinking water with spectinomycin and lincomycin-spectinomycin in experimental Escherichia coli infection in poultry. Vet. Q. 1988;10(3):191-197. doi: 10.1080/01652176.1988.9694170 | Include |
| 117 | Labarthe JC, Guillot JF, Mouline C, Bree A. Etude quantitative de l'effet antibacterien du cefotaxime et de la ceftriaxone lors de bacteriemie experiementales a Escherichia coli chez le poulet. Pathol. Biol. 1989;37(5 Pt 2):664-667. | Include |
| 118 | Phukan A, Kalita CC, Dutta GN. Treatment of experimental colibacillosis in chickens. Indian J. Vet. Med. 1989;9(2):139-141. | Include |
| 119 | Mogenet L, Bezille P, Guyonnet J, Karembe H. Comparaison de la flumequine (Flumisol) a l'amoxicilline (Vetrimox poudre orale) dans deux modes d'administration par voie orale, en traitement de la colibacillose du poulet: approche pharmacodynamique et Clinique. Revue Med. Vet. 1997;148(10):793-804. | Include |
| 120 | Charleston B, Gate JJ, Aitken IA, Stephan B, Froyman R. Comparison of the efficacies of three fluoroquinolone antimicrobial agents, given as continuous or pulsed-water medication, against Escherichia coli infection in chickens. Antimicrob. Agents Chemother. 1998;42(1):83-87. doi: 10.1128/AAC.42.1.83 | Include |
| 121 | Sumano H, Ocampo L, Azuara J. Antibacterial activity, pharmacokinetics and therapeutic efficacy in poultry of a new cephalosporin-fluoroquinolone (CQ) molecule. J. Appl. Anim. Res. 1998;13(1/2):169-178. doi: 10.1080/09712119.1998.9706683 | Include |
| 122 | Ashraf M, Arif Q, Khan KA. Efficacy of gentamicin after intrayolk administration in experimentally induced omphalitis in broiler chicks. Pakistan Vet. J. 2002;22:197-198. | Include |
| 123 | Chansiripornchai N, Sasipreeyajan J. Efficacy of sarafloxacin in broilers after experimental infection with Escherichia coli. Vet. Res. Commun. 2002;26(4):255-262. doi: 10.1023/a:1016078222398 | Include |
| 124 | Fernandez A, Lara C, Loste A, Marca MC. Efficacy of calcium fosfomycin for the treatment of experimental infection of broiler chicken with Escherichia coli O78:K80. Vet. Res. Commun. 2002;26(6):427-436. doi: 10.1023/a:1020582207129 | Include |
| 125 | Sarközy G, Semjen G, Laczay P, Horvath E, Schmidt J. Pulse and continued oral norfloxacin treatment of experimentally induced Escherichia coli infection in broiler chicks and turkey poults. Acta Vet. Hung. 2002;50(2):199-210. doi: 10.1556/AVet.50.2002.2.9 | Include |
| 126 | Glisson JR, Hofacre CL, Mathis GF. Comparative efficacy of enrofloxacin, oxytetracycline, and sulfadimethoxine for the control of morbidity and mortality caused by Escherichia coli in broiler chickens. Avian Dis. 2004;48(3):658-662. doi: 10.1637/7166 | Include |
| 127 | Huff WE, Huff GR, Rath NC, Balog JM, Donoghue AM. Therapeutic efficacy of bacteriophage and Baytril (Enrofloxacin) individually and in combination to treat colibacillosis in broilers. Poult. Sci. 2004;83(12):1944-1947. doi: 10.1093/ps/83.12.1944 | Include |
| 128 | Velkers FC, Loo AJH, Madin F, van Eck JHH. Isopathic and pluralist homeopathic treatment of commercial broilers with experimentally induced colibacillosis. Res. Vet. Sci. 2005;78(1):77-83. doi: 10.1016/j.rvsc.2004.06.005 | Include |
| 129 | Phad VN, Moregaonkar SD, Bhonsale AV, Khan MA, Markandeya NM. Efficacy of Colis-V in alleviating pathological alterations caused by experimental colibacillosis in broilers. Indian J. Vet. Pathol. 2007;31(2):181-182. | Include |
| 130 | Masoud EA, AI-Dana H, Mancruos EE, Alam H. Efficacy of apramycin in control of E. coli infection in broilers. Vet. Med. J. 2008;10(2):97-107. | Include |
| 131 | Chansiripornchai N. Comparative efficacy of enrofloxacin and oxytetracycline by different administration methods in broilers after experimental infection with Avian Pathogenic Escherichia coli. Thai J. Vet. Med. 2009;39(3):231-236. | Include |
| 132 | Abd El-Ghany WA, Madian K. Control of experimental colisepticaemia in broiler chickens using sarafloxacin. Life Sci. J. 2011;8(3):318-328. | Include |
| 133 | Dheilly A, Bouder A, Le Devendec L, Hellard G, Kempf I. Clinical and microbial efficacy of antimicrobial treatments of experimental avian colibacillosis. Vet. Microbiol. 2011;149(3-4):422-429. doi: 10.1016/j.vetmic.2010.11.033 | Include |
| 134 | Hassan ER, Mahgoob KM, ELbayoumi Zeinab KM, Amin Girh MS, Mekky HM. Comparative studies between the effects of antibiotic (oxytetracycline); probiotic and acidifier on E. coli infection and immune response in broiler chickens. J. American Sci. 2012;8(4):795-801. | Include |
| 135 | Peek HW, Halkes SBA, Tomassen MMM, Mes JJ, Landman WJM. In vivo screening of five phytochemicals/extracts and a fungal immunomodulatory protein against colibacillosis in broilers. Avian Pathol. 2013;42(3):235-247. doi: 10.1080/03079457.2013.780121 | Include |
| 136 | Abd El-Ghany WA, Ismail M. Tackling experimental colisepticaemia in broiler chickens using phytobiotic essential oils and antibiotic alone or in combination. Iranian J. Vet. Res. 2014;15(2):110-115. doi: 10.22099/ijvr.2014.2341 | Include |
| 137 | Shalaby NA, Belih SS, Ragab AM, EL-Hadad SF. Influence of chitonal on some hematological, biochemical, immunological parameters and histopathological changes in broilers experimentally infected with E. coli. Glob. J. Agric. Food Safety Sci. 2014;1:454-472. | Include |
| 138 | Lezzar N, Bensari C, Lezzar A, Smati F, Benlabed K, Bentchouala C, et al. Effect of oral treatment flumequine on an experimental colibacillosis on chicken of Flesh (Algeria). Global Vet. 2015;14(4):608-612. | Include |
| 139 | Foltz KL, Ritzi MM, Barrett NW, Evans NP, Collins D, Sriranganathan N, et al. Efficacy of Lactobacillus plantarum supplementation in broilers challenged with avian pathogenic Escherichia coli and Salmonella Typhimurium. J. Appl. Poult. Res. 2017;26(3):316-324. doi: 10.3382/japr/pfw074 | Include |
| 140 | El-Keredy AMS, Barakat M, Gehan IEA, Nehal NAA, Salim AA. Effect of some antibiotic alternatives on experimentally Escherichia coli infected broiler chicks. Alexandria J. Vet. Sci. 2019;63(1):101-115. | Include |
| 141 | Roth N, Hofacre C, Zitz U, Mathis GF, Moder K, Doupovec B, et al. Prevalence of antibiotic-resistant E. coli in broilers challenged with a multi-resistant E. coli strain and received ampicillin, an organic acid-based feed additive or a synbiotic preparation. Poult. Sci. 2019;98(6):1-10. doi: 10.3382/ps/pez004 | Include |
| 142 | Aguilar-Urquizo E, Itza-Ortiz MF, Sangines-Garcia JR, Pineiro-Vazquez AT, Reyes-Ramirez A, Pinacho-Santana B. Phytobiotic activity of Piper auritum and Ocimum basilicum on avian E. coli. Braz. J. Poult. Sci. 2020;22(1):1-10. doi: 10.1590/1806-9061-2019-1167 | Include |
| 143 | Ahmad T, Muhammad G, Sharif A, Nadeem M, Shakoor A, Rizwan M. Survey of antibiotic treatment of Escherichia coli infection in broilers and efficacy of enrofloxacin plus colistin in experimental colibacillosis. Pure Appl. Biol. 2020;9(3):1864-1872. doi: 10.19045/bspab.2020.90199 | Include |
| 144 | Tarazi YH, Abu-Basha EA, Ismail ZB, Tailony RA. In vitro and in vivo efficacy study of cefepime, doripenem, tygecycline, and tetracycline against extended-spectrum beta-lactamases Escherichia coli in chickens. Vet. World. 2020;13(3):446-451. doi: 10.14202/vetworld.2020.446-451 | Include |
| 145 | ul Haq I, Hafeez A, Khan RU. Protective effect of Nigella sativa and Saccharomyces cerevisiae on zootechnical characteristics, fecal Escherichia coli and hematopoietic potential in broiler infected with experimental colibacillosis. Livestock Sci. 2020;239:104119. doi: 10.1016/j.livsci.2020.104119 | Include |
| 146 | El Hammed WA, Soufy H, El-Shemy A, Nasr SM, Dessouky MI. Treatment effect of Oregano essential oil in broiler chickens experimentally infected with Avian Pathogenic Escherichia coli O27 with emphasis on hemogram, serum biochemistry and histopathology of vital organs. Egypt J. Chem. 2021;64(10):6105-6122. doi: 10.21608/EJCHEM.2021.86333.4181 | Include |
| 147 | Jahanian E, Mahdavi AH, Jahanian R. Silymarin improved the growth performance via modulating the microbiota and mucosal immunity in Escherichia coli-challenged broiler chicks. Livestock Sci. 2021;249:104529. doi: 10.1016/j.livsci.2021.104529 | Include |
| 148 | Ahmad T, Fiaz M, Sharif A, Nadeem M, Sajjad-ur-Rehman, Rizwan M, Umer M. In vitro and in vivo evaluation of antimicrobials in Escherichia coli infection in broilers and evaluation of ciprofloxacin in induced colibacillosis. Pure Appl. Biol. 2022;11(3):744-754. doi: 10.19045/bspab.2022.110075 | Include |
| 149 | Eid S, Tolba HMN, Hamed RI, Al-Atfeehy NM. Bacteriophage therapy as an alternative biocontrol against emerging multidrug resistant E. coli in broilers. Saudi J. Biol. Sci. 2022;29:3380-3389. doi: 10.1016/j.sjbs.2022.02.015 | Include |
| 150 | El-Tahawy AO, Said AA, Shams GA, Hassan HM, Hassan AM, Amer SA, El-Nabtity SM. Evaluation of cefquinome’s efficacy in controlling avian colibacillosis and detection of its residues using high performance liquid chromatography (HPLC). Saudi J. Biol. Sci. 2022;29(5):3502-3510. doi: 10.1016/j.sjbs.2022.02.029 | Include |
| 151 | Gunawardana T, Ahmed KA, Popowich S, Kurukulasuriya S, Lockerbie B, Karunarathana R, et al. Comparison of therapeutic antibiotics, probiotics, and synthetic CpG-ODNs for protective efficacy against Escherichia coli lethal infection and impact on the immune system in neonatal broiler chickens. Avian Dis. 66:165-175. doi: 10.1637/aviandiseases-D-22-00011 | Include |
| 152 | Helmy YA, Kathayat D, Galgozy GC Jr K, Fuchs JM, Rajashekara G. Efficacy of quorum sensing and growth inhibitors alone and in combination against avian pathogenic Escherichia coli infection in chickens. Poult. Sci. 2023;102:102543. doi: 10.1016/j.psj.2023.102543 | Include |
| 153 | Ahmad T, Fiaz M, Sharif A, Nadeem M, Sajjad-ur-Rehman, Rizwan M, Umer M. In vitro and in vivo evaluation of antimicrobials in Escherichia coli infection in broilers and evaluation of ciprofloxacin in induced colibacillosis. Pure Appl. Biol. 2022;11(3):744-754. doi: 10.19045/bspab.2022.110075 | Duplicate |
| 154 | Danielescu, N., S. Ghergariu, N. Slavcovici, and G. Giurgiu. "Epidemiology of an outbreak of colibacillosis in chickens treated with Comaciclin (combination of tetracycline, erythromycin and trisodium citrate)." (1988): 641-644. | Not english, french or spanish (Romanian) |
| 155 | Nicolas M, Trotereau A, Culot A, Moodley A, Atterbury R, Wagemans J, Lavigne R, Velge P, Schouler C. Isolation and characterization of a novel phage collection against avian-pathogenic Escherichia coli. Microbiology Spectrum. 2023 Jun 15;11(3):e04296-22. | Not concerning broilers |
| 156 | Mogenet L, Bezille P, Guyonnet J, Karembe H. Comparaison de la flumequine (flumisol) a l'amoxicilline (vetrimoxin poudre orale) dans deux modes d'administration par voie orale, en traitement de la colibacillose du poulet: approche pharmacodynamique et clinique. Revue de Medecine Veterinaire (France). 1997;148(10). | Duplicate |
